# Supplementary material for: The effects of social determinants on children’s health outcomes in Bangladesh slums through an intersectionality lens: An application of multilevel analysis of individual heterogeneity and discriminatory accuracy (MAIHDA)
Source: PLOS Glob Public Health. 2023 Mar 8;3(3):e0001588. doi: 10.1371/journal.pgph.0001588 (PMC10022045; doi:10.1371/journal.pgph.0001588)
Supplement: S1 File — (DOCX) [file pgph.0001588.s001.docx]

**S1 File. Statistical details**

Let$y_{ij}$ denote a binary health outcome (i.e., whether child had an illness or not) for child $i\left( i=1,\ldots,n \right)$ in intersectional strata $j \left( j=1,\ldots,N \right)$ where,

$y_{ij}=\left\{ \begin{aligned} 0 absence of health outcome \\ 1 Presence of health outcome \end{aligned} \right.$ ……………. Equation (1)

$y_{ij}$ is assumed to follow a Bernoulli distribution, with probabilities $\pi_{ij}=Pr\left( y_{ij}=0 \right)$ the probability of child $i$ from intersectional stratum$j$having no health outcome and $1-\pi_{ij}=Pr\left( y_{i}=1 \right)$the probability of child $i$ from stratus$j$having a health outcome. Let $X_{ij}^{'}$ be a vector of social determinants of health (SDoH) used as explanatory variables. The multilevel logistic for Model 1 with no main effects takes the form

$logit\left( \pi_{ij} \right)=log\left( \frac{\pi_{ij}}{1-\pi_{ij}} \right)=\beta_{0}+\mu_{0j}\ldots\ldots\ldots\ldots\ldots. Equation (2)$

where $\beta_{0}$ is the intercept and $\mu_{0j}\sim N\left( 0,\sigma_{\mu}^{2} \right)$ represents the random intercept for the intersectional stratum level residual which is normally distributed with mean $0$ and variance $\sigma_{\mu}^{2}$. Model 1 includes explanatory variables, so the intersectional stratum random effect captures both the main effects of SDoH used to define intersectional strata and their interactions. Assuming no omitted variable bias, the intersectional strata level residual $\mu_{0j}$ captures the unique interaction effect for each intersectional strata (i.e., intersectional -specific differences in health condition) while accounting for sample size differences for each social group.

Eq. 2 can be extended into Model 2 by including main effects (i.e., SDoH used in construction intersectional strata) as explanatory variables and takes the form

$logit\left( \pi_{ij} \right)=log\left( \frac{\pi_{ij}}{1-\pi_{ij}} \right)=\beta_{0}+X_{ij}^{'}\beta+\mu_{0j}$……………. Equation (3)

where $\beta_{0}$is the intercept, $X_{ij}^{'}$is a vector of vector of SDoH used in creating intersectional strata with coefficient vector $\beta$ , and $\mu_{0j}\sim N\left( 0,\sigma_{\mu}^{2} \right)$ is a random intercept assumed to follow a normal distribution with mean $0$ and variance $\sigma_{\mu}^{2}.$

We used variance partition coefficient (VPC) to estimate discriminatory accuracy of intersectional strata n Models 1 and Model 2 [1,2]. VPC indicates the share of the total individual variance in the probability of having a health condition that is accounted for at the intersectional strata level [1]. VPCs were calculated for both Model 1 and Model 2 using Equation (4):

$VPC=\left( \frac{\sigma_{\mu}^{2}}{\sigma_{\mu}^{2}+ 3.29} \right)\times100\%$ ……………. Equation (4)

Where $\sigma_{\mu}^{2}$ denotes the between stratum variance in the propensity for having a health condition, while 3.29 indicates the within stratum between individual stratum variance constrained equal to the variance of the standard logistic distribution [3]. VPC has been presented as the percentage share of individual variance which lies between strata. In model 2, assuming no relevant variables were omitted when constructing strata, a high VPC informs on the existence of intersectional multiplicative interaction effects [2,4,5].

The proportion of variance explained by the adding main effects is estimated by calculating the proportional change in variance (PCV) of intersectional strata between null model and model including main effects [2].

$PCV=\left( \frac{\sigma_{\mu(1)}^{2}-\sigma_{\mu(2)}^{2}}{\sigma_{\mu(1)}^{2}} \right)\times100\%$ ……………. Equation (5)

where $\sigma_{\mu(1)}^{2}$ and $\sigma_{\mu(2)}^{2}$represents the intersectional strata variances in the null model and the model containing main effects respectively. The PCV represents the proportion of the total between-stratum variance of intersectional strata of the null model that is explained after including main effects. In the absence of any stratum specific interactions, the main effects used to construct the intersectional strata would completely explain the between stratum variance and all stratum random effects would be equal to zero. This implies that, the lower the PCV, the higher the amount explained variance which can be due to interaction effects or to omitted variable bias [2,4,5].

**References**

1. Goldstein H, Browne W, Rasbash J. Partitioning Variation in Multilevel Models. Understanding Statistics [Internet]. 2002 [cited 2021 Dec 3];1(4):223–31. Available from: http://www.tandfonline.com/doi/abs/10.1207/S15328031US0104_02

2. Merlo J, Chaix B, Ohlsson H, Beckman A, Johnell K, Hjerpe P, et al. A brief conceptual tutorial of multilevel analysis in social epidemiology: using measures of clustering in multilevel logistic regression to investigate contextual phenomena. J Epidemiol Community Health. 2006;60(4):290–7.

3. Goldstein H. Multilevel Statistical Models. John Wiley & Sons; 2011. 376 p.

4. Evans CR, Leckie G, Merlo J. Multilevel versus single-level regression for the analysis of multilevel information: The case of quantitative intersectional analysis. Social Science & Medicine [Internet]. 2020 [cited 2021 Dec 3];245:112499. Available from: https://linkinghub.elsevier.com/retrieve/pii/S0277953619304927

5. Persmark A, Wemrell M, Zettermark S, Leckie G, Subramanian SV, Merlo J. Precision public health: Mapping socioeconomic disparities in opioid dispensations at Swedish pharmacies by Multilevel Analysis of Individual Heterogeneity and Discriminatory Accuracy (MAIHDA). PLoS One [Internet]. 2019;14(10):e0224008. Available from: https://journals.plos.org/plosone/article?id=10.1371/journal.pone.0220322
