# Supplementary material for: The effects of social determinants on children’s health outcomes in Bangladesh slums through an intersectionality lens: An application of multilevel analysis of individual heterogeneity and discriminatory accuracy (MAIHDA)
Source: PLOS Glob Public Health. 2023 Mar 8;3(3):e0001588. doi: 10.1371/journal.pgph.0001588 (PMC10022045; doi:10.1371/journal.pgph.0001588)
Supplement: S1 Table — (DOCX) [file pgph.0001588.s002.docx]

**S1 Table. Description of health outcomes and predictor variables for the analysis for Bangladesh Urban Health Survey 2013 (UHS 2013)**

| Variable name | Description | Categories |
| --- | --- | --- |
| Health outcomes | | |
| Acute Respiratory Infections (ARI) | In UHS 2013, ARI is defined as cough accompanied by short, rapid, or difficult breathing which is chest related. It is considered as proxy pneumonia | Yes |
|  |  | No |
| Fever | Fever is defined as whether a child had fever or not in the two weeks preceding the survey. | Yes |
|  |  | No |
| Cough | Whether a child had cough or not in the two weeks preceding the survey | Yes |
|  |  | No |
| Predictors | | |
| Children demographic characteristics | | |
| Age | Children up to five were categorised into two groups: “Up to 1 year old (Infants)” and “2-5 years”. | 1 year and less (infants) |
|  |  | 2 -5 years |
| Sex | Sex of a child coded as either male or female | Male |
|  |  | Female |
| Women characteristics | | |
| Age | Mothers ’age was categorised into two groups: “18 years and less (<18)” and “19 years and above. Note that the legal age of women at first marriage is 18 years in Bangladesh. | 18 years and under |
|  |  | 19 years and above |
| Ever attended school | Mother ever attended school | Yes |
|  |  | No |
| Marital status | Mother’s marital status was categorised into two groups: “Being married”, “Not being married” | Being married |
|  |  | Not being married |
| Employment | Mothers of respective children was employed last 12 months. | Yes |
|  |  | No |
| Religion | Mother’s religion was categorized into two groups: “Islam”, “Minority religion”. Note that Buddhism, Hinduism, Christianism were combined as minority religion in this study. | Islam |
|  |  | Minority religion |
| Head of household demographic characteristics | | |
| Gender | Sex of head of household was coded as either male or female | Female |
|  |  | Male |
| Age | Age of head of household was categorised into three categories. | 17 – 24years |
|  |  | 25 -34 years |
|  |  | 35 years above |
| Marital status | Head of household’s marital status was categorised into two groups: “Being married”, “Not being married” | None |
|  |  | educated |
|  |  | Don’t know and not applicable |
| Social structure | | |
| Wealth index | Wealth Index were regrouped from five (categories) to three by combining poorest and poorer into one group “poor” and richer and richest into one group “rich” and “Middle”. | Rich |
|  |  | Middle |
|  |  | Poor |
| Length of stay | The number of years lived in slums by the household grouped into new migrants (i.e.,2 years and less), old migrants (i.e., more than 2 years), and not applicable/missing. | New migrants |
|  |  | Old migrants |
|  |  | Not applicable |
| Cooking Fuel used in household | Cooking fuels used in the household were categorized into four: “Charcoal, dung cakes etc.”, “Kerosene or liquid gas”, “Natural gas”, and “Wood fuel” | Charcoal, dung cakes etc. |
|  |  | Kerosene or liquid gas |
|  |  | Natural gas |
|  |  | Wood fuel |
| Garbage disposal method of households | Garbage disposal method of households were categorized into four: “Disposed within premises”, “Collected from home”, “Disposed in bin outside”, and “Disposed in open spaces” | Disposed within premises |
|  |  | Collected from home |
|  |  | Disposed in bin outside |
|  |  | Disposed in open spaces |
| Migration status of households | Household’s availability of any food in the last 12 months |  |
|  |  |  |
| Housing Type | Housing type was categorized into two groups: “Multiple story” and “Single story”. We collapsed *Jhupri, Mess* as single story. | Multiple story |
|  |  | Single story |
| Ownership of the dwelling | Two categories: “yes”, “no” | No |
|  |  | Yes |
| Ownership of the land | Two categories: “yes”, “no” | No |
|  |  | Yes |
| Having Separate kitchen | Two categories: “yes”, “no” | No |
|  |  | Yes |
| Division | Before 2015, there were 7 administrative divisions in Bangladesh. To reduce categories, we combined “Barisal”, “Chittagong”, “Rangpur”, and “Sylhet” division and named them as “Others division” . So, there are four categories as “Dhaka”, “Khulna”, “Rajshahi” and “Others division”. ARISE Bangladesh Team conducts research in Dhaka, Rajshahi and Khulna divisions. | Others division |
|  |  | Dhaka |
|  |  | Khulna |
|  |  | Rajshahi |
