# Supplementary material for: The effects of social determinants on children’s health outcomes in Bangladesh slums through an intersectionality lens: An application of multilevel analysis of individual heterogeneity and discriminatory accuracy (MAIHDA)
Source: PLOS Glob Public Health. 2023 Mar 8;3(3):e0001588. doi: 10.1371/journal.pgph.0001588 (PMC10022045; doi:10.1371/journal.pgph.0001588)
Supplement: S2 Table — (DOCX) [file pgph.0001588.s003.docx]

**S2 Table. Distribution of socio determinants characteristics for cough**

| Variable | Category | Cough | | Overall |
| --- | --- | --- | --- | --- |
|  |  | **No** | **Yes** |  |
| n |  | 2562 | 611 | 3173 |
| U5y demographic characteristics |  |  |  |  |
| Children sex (%) | Female | 1257 (49.1) | 289 (47.3) | 1546 (48.7) |
|  | Male | 1305 (50.9) | 322 (52.7) | 1627 (51.3) |
| Children age (%) | 1 year and less | 898 (35.1) | 266 (43.5) | 1164 (36.7) |
|  | 2 to 5 years | 1664 (64.9) | 345 (56.5) | 2009 (63.3) |
| Mothers’ sociodemographic characteristics |  |  |  |  |
| Mothers age (%) | <18 years | 67 (2.6) | 25 (4.1) | 92 (2.9) |
|  | 18 years and above | 2495 (97.4) | 586 (95.9) | 3081 (97.1) |
| Mothers’ religion (%) | Islam | 2419 (94.4) | 568 (93.0) | 2987 (94.1) |
|  | Minority religion | 143 (5.6) | 43 (7.0) | 186 (5.9) |
| Mother ever attended school (%) | No | 554 (21.6) | 120 (19.6) | 674 (21.2) |
|  | Yes | 2008 (78.4) | 491 (80.4) | 2499 (78.8) |
| Mothers’ employment (%) | No | 1931 (75.4) | 492 (80.5) | 2423 (76.4) |
|  | Yes | 631 (24.6) | 119 (19.5) | 750 (23.6) |
| Mother marital status (%) | Married | 2509 (97.9) | 593 (97.1) | 3102 (97.8) |
|  | Not married | 53 (2.1) | 18 (2.9) | 71 (2.2) |
| Head of household sociodemographic characteristics |  |  |  |  |
| Age (%) | 13 - 29 years | 1133 (44.2) | 311 (50.9) | 1444 (45.5) |
|  | 30 - 44 years | 1076 (42.0) | 203 (33.2) | 1279 (40.3) |
|  | 45 years and above | 353 (13.8) | 97 (15.9) | 450 (14.2) |
| Sex (%) | Female | 217 (8.5) | 53 (8.7) | 270 (8.5) |
|  | Male | 2345 (91.5) | 558 (91.3) | 2903 (91.5) |
| Marital status (%) | Married | 2468 (96.3) | 578 (94.6) | 3046 (96.0) |
|  | Currently not married | 94 (3.7) | 33 (5.4) | 127 (4.0) |
| Social structure characteristics of the household |  |  |  |  |
| Wealth index (%) | Rich | 242 (9.4) | 62 (10.1) | 304 (9.6) |
|  | Middle | 545 (21.3) | 126 (20.6) | 671 (21.1) |
|  | Poor | 1775 (69.3) | 423 (69.2) | 2198 (69.3) |
| Housing condition (%) | Multiple story | 229 (8.9) | 57 (9.3) | 286 (9.0) |
|  | Single story | 2333 (91.1) | 554 (90.7) | 2887 (91.0) |
| Having separate kitchen (%) | No | 1502 (58.6) | 333 (54.5) | 1835 (57.8) |
|  | Yes | 1060 (41.4) | 278 (45.5) | 1338 (42.2) |
| Cooking fuel used (%) | Charcoal, dung cakes, etc. | 70 (2.7) | 25 (4.1) | 95 (3.0) |
|  | Kerosene or liquid gas | 46 (1.8) | 11 (1.8) | 57 (1.8) |
|  | Natural gas | 1654 (64.6) | 387 (63.3) | 2041 (64.3) |
|  | Wood fuel | 792 (30.9) | 188 (30.8) | 980 (30.9) |
| Migration status (%) | Old migrants | 1949 (76.1) | 448 (73.3) | 2397 (75.5) |
|  | New migrants | 613 (23.9) | 163 (26.7) | 776 (24.5) |
| Administrative division (%) | Dhaka | 1762 (68.8) | 346 (56.6) | 2108 (66.4) |
|  | Khulna | 117 (4.6) | 36 (5.9) | 153 (4.8) |
|  | Rajshahi | 40 (1.6) | 16 (2.6) | 56 (1.8) |
|  | Other divisions | 643 (25.1) | 213 (34.9) | 856 (27.0) |
| Garbage disposal methods (%) | Disposed within premises | 119 (4.6) | 48 (7.9) | 167 (5.3) |
|  | Collected from home | 906 (35.4) | 188 (30.8) | 1094 (34.5) |
|  | Disposed in bin outside | 322 (12.6) | 82 (13.4) | 404 (12.7) |
|  | Disposed in open spaces | 1215 (47.4) | 293 (48.0) | 1508 (47.5) |
| Ownership of dwelling (%) | Employer or other | 75 (2.9) | 18 (2.9) | 93 (2.9) |
|  | Owned | 413 (16.1) | 116 (19.0) | 529 (16.7) |
|  | Rented | 2072 (80.9) | 477 (78.1) | 2549 (80.3) |
|  | NA | 2 (0.1) | 0 (0.0) | 2 (0.1) |
| Ownership of land (%) | Government | 144 (5.6) | 37 (6.1) | 181 (5.7) |
|  | Landlord | 2049 (80.0) | 468 (76.6) | 2517 (79.3) |
|  | NGO | 21 (0.8) | 11 (1.8) | 32 (1.0) |
|  | Respondent or another resident | 346 (13.5) | 95 (15.5) | 441 (13.9) |
|  | NA | 2 (0.1) | 0 (0.0) | 2 (0.1) |
