# Supplementary material for: The effects of social determinants on children’s health outcomes in Bangladesh slums through an intersectionality lens: An application of multilevel analysis of individual heterogeneity and discriminatory accuracy (MAIHDA)
Source: PLOS Glob Public Health. 2023 Mar 8;3(3):e0001588. doi: 10.1371/journal.pgph.0001588 (PMC10022045; doi:10.1371/journal.pgph.0001588)
Supplement: S3 Table — (DOCX) [file pgph.0001588.s004.docx]

**S3 Table. Distribution of socio determinants characteristics for fever**

| Variable | Category | Fever | | Overall |
| --- | --- | --- | --- | --- |
|  |  | **No** | **Yes** |  |
| n |  | 2276 | 907 | 3183 |
| Children’s demographic characteristics |  |  |  |  |
| Children sex (%) | Female | 1116 (49.0) | 435 (48.0) | 1551 (48.7) |
|  | Male | 1160 (51.0) | 472 (52.0) | 1632 (51.3) |
| Children age (%) | 1 year and less | 802 (35.2) | 365 (40.2) | 1167 (36.7) |
|  | 2 to 5 years | 1474 (64.8) | 542 (59.8) | 2016 (63.3) |
| Mothers’ sociodemographic characteristics |  |  |  |  |
| Mothers age (%) | <18 years | 57 (2.5) | 36 (4.0) | 93 (2.9) |
|  | 18 years and above | 2219 (97.5) | 871 (96.0) | 3090 (97.1) |
| Mothers’ religion (%) | Islam | 2140 (94.0) | 857 (94.5) | 2997 (94.2) |
|  | Minority religion | 136 (6.0) | 50 (5.5) | 186 (5.8) |
| Mother ever attended school (%) | No | 504 (22.1) | 172 (19.0) | 676 (21.2) |
|  | Yes | 1772 (77.9) | 735 (81.0) | 2507 (78.8) |
| Mothers’ employment (%) | No | 1714 (75.3) | 716 (78.9) | 2430 (76.3) |
|  | Yes | 562 (24.7) | 191 (21.1) | 753 (23.7) |
| Mother marital status (%) | Married | 2228 (97.9) | 883 (97.4) | 3111 (97.7) |
|  | Not married | 48 (2.1) | 24 (2.6) | 72 (2.3) |
| Head of household sociodemographic characteristics |  |  |  |  |
| Age (%) | 13 - 29 years | 979 (43.0) | 471 (51.9) | 1450 (45.6) |
|  | 30 - 44 years | 978 (43.0) | 304 (33.5) | 1282 (40.3) |
|  | 45 years and above | 319 (14.0) | 132 (14.6) | 451 (14.2) |
| Sex (%) | Female | 196 (8.6) | 75 (8.3) | 271 (8.5) |
|  | Male | 2080 (91.4) | 832 (91.7) | 2912 (91.5) |
| Marital status (%) | Married | 2189 (96.2) | 866 (95.5) | 3055 (96.0) |
|  | Currently not married | 87 (3.8) | 41 (4.5) | 128 (4.0) |
| Social structure characteristics of the household |  |  |  |  |
| Wealth index (%) | Rich | 222 (9.8) | 84 (9.3) | 306 (9.6) |
|  | Middle | 505 (22.2) | 169 (18.6) | 674 (21.2) |
|  | Poor | 1549 (68.1) | 654 (72.1) | 2203 (69.2) |
| Housing condition (%) | Multiple story | 204 (9.0) | 84 (9.3) | 288 (9.0) |
|  | Single story | 2072 (91.0) | 823 (90.7) | 2895 (91.0) |
| Having separate kitchen (%) | No | 1350 (59.3) | 490 (54.0) | 1840 (57.8) |
|  | Yes | 926 (40.7) | 417 (46.0) | 1343 (42.2) |
| Cooking fuel used (%) | Charcoal, dung cakes, etc. | 65 (2.9) | 31 (3.4) | 96 (3.0) |
|  | Kerosene or liquid gas | 43 (1.9) | 14 (1.5) | 57 (1.8) |
|  | Natural gas | 1483 (65.2) | 565 (62.3) | 2048 (64.3) |
|  | Wood fuel | 685 (30.1) | 297 (32.7) | 982 (30.9) |
| Migration status (%) | Old migrants | 1734 (76.2) | 670 (73.9) | 2404 (75.5) |
|  | New migrants | 542 (23.8) | 237 (26.1) | 779 (24.5) |
| Administrative division (%) | Dhaka | 1574 (69.2) | 543 (59.9) | 2117 (66.5) |
|  | Khulna | 108 (4.7) | 45 (5.0) | 153 (4.8) |
|  | Rajshahi | 36 (1.6) | 20 (2.2) | 56 (1.8) |
|  | Other divisions | 558 (24.5) | 299 (33.0) | 857 (26.9) |
| Garbage disposal methods (%) | Disposed within premises | 107 (4.7) | 60 (6.6) | 167 (5.2) |
|  | Collected from home | 819 (36.0) | 278 (30.7) | 1097 (34.5) |
|  | Disposed in bin outside | 291 (12.8) | 115 (12.7) | 406 (12.8) |
|  | Disposed in open spaces | 1059 (46.5) | 454 (50.1) | 1513 (47.5) |
| Ownership of dwelling (%) | Employer or other | 69 (3.0) | 24 (2.6) | 93 (2.9) |
|  | Owned | 368 (16.2) | 164 (18.1) | 532 (16.7) |
|  | Rented | 1837 (80.7) | 719 (79.3) | 2556 (80.3) |
|  | NA | 2 (0.1) | 0 (0.0) | 2 (0.1) |
| Ownership of land (%) | Government | 123 (5.4) | 58 (6.4) | 181 (5.7) |
|  | Landlord | 1820 (80.0) | 704 (77.6) | 2524 (79.3) |
|  | NGO | 18 (0.8) | 14 (1.5) | 32 (1.0) |
|  | Respondent or another resident | 313 (13.8) | 131 (14.4) | 444 (13.9) |
|  | NA | 2 (0.1) | 0 (0.0) | 2 (0.1) |
