# Supplementary material for: The effects of social determinants on children’s health outcomes in Bangladesh slums through an intersectionality lens: An application of multilevel analysis of individual heterogeneity and discriminatory accuracy (MAIHDA)
Source: PLOS Glob Public Health. 2023 Mar 8;3(3):e0001588. doi: 10.1371/journal.pgph.0001588 (PMC10022045; doi:10.1371/journal.pgph.0001588)
Supplement: S4 Table — (DOCX) [file pgph.0001588.s005.docx]

**S4 Table. Distribution of socio determinants characteristics for acute respiratory infections (ARI)**

| Variable | Category | ARI | | Overall |
| --- | --- | --- | --- | --- |
|  |  | **No** | **Yes** |  |
| n |  | 3024 | 159 | 3183 |
| Children’s demographic characteristics |  |  |  |  |
| Children sex (%) | Female | 1475 (48.8) | 76 (47.8) | 1551 (48.7) |
|  | Male | 1549 (51.2) | 83 (52.2) | 1632 (51.3) |
| Children age (%) | 1 year and less | 1094 (36.2) | 73 (45.9) | 1167 (36.7) |
|  | 2 to 5 years | 1930 (63.8) | 86 (54.1) | 2016 (63.3) |
| Mothers’ sociodemographic characteristics |  |  |  |  |
| Mothers age (%) | <18 years | 85 (2.8) | 8 (5.0) | 93 (2.9) |
|  | 18 years and above | 2939 (97.2) | 151 (95.0) | 3090 (97.1) |
| Mothers’ religion (%) | Islam | 2844 (94.0) | 153 (96.2) | 2997 (94.2) |
|  | Minority religion | 180 (6.0) | 6 (3.8) | 186 (5.8) |
| Mother ever attended school (%) | No | 648 (21.4) | 28 (17.6) | 676 (21.2) |
|  | Yes | 2376 (78.6) | 131 (82.4) | 2507 (78.8) |
| Mothers’ employment (%) | No | 2298 (76.0) | 132 (83.0) | 2430 (76.3) |
|  | Yes | 726 (24.0) | 27 (17.0) | 753 (23.7) |
| Mother marital status (%) | Married | 2954 (97.7) | 157 (98.7) | 3111 (97.7) |
|  | Not married | 70 (2.3) | 2 (1.3) | 72 (2.3) |
| Head of household sociodemographic characteristics |  |  |  |  |
| Age (%) | 13 - 29 years | 1361 (45.0) | 89 (56.0) | 1450 (45.6) |
|  | 30 - 44 years | 1241 (41.0) | 41 (25.8) | 1282 (40.3) |
|  | 45 years and above | 422 (14.0) | 29 (18.2) | 451 (14.2) |
| Sex (%) | Female | 251 (8.3) | 20 (12.6) | 271 (8.5) |
|  | Male | 2773 (91.7) | 139 (87.4) | 2912 (91.5) |
| Marital status (%) | Married | 2906 (96.1) | 149 (93.7) | 3055 (96.0) |
|  | Currently not married | 118 (3.9) | 10 (6.3) | 128 (4.0) |
| Social structure characteristics of the household |  |  |  |  |
| Wealth index (%) | Rich | 286 (9.5) | 20 (12.6) | 306 (9.6) |
|  | Middle | 646 (21.4) | 28 (17.6) | 674 (21.2) |
|  | Poor | 2092 (69.2) | 111 (69.8) | 2203 (69.2) |
| Housing condition (%) | Multiple story | 271 (9.0) | 17 (10.7) | 288 (9.0) |
|  | Single story | 2753 (91.0) | 142 (89.3) | 2895 (91.0) |
| Having separate kitchen (%) | No | 1746 (57.7) | 94 (59.1) | 1840 (57.8) |
|  | Yes | 1278 (42.3) | 65 (40.9) | 1343 (42.2) |
| Cooking fuel used (%) | Charcoal, dung cakes, etc. | 88 (2.9) | 8 (5.0) | 96 (3.0) |
|  | Kerosene or liquid gas | 53 (1.8) | 4 (2.5) | 57 (1.8) |
|  | Natural gas | 1949 (64.5) | 99 (62.3) | 2048 (64.3) |
|  | Wood fuel | 934 (30.9) | 48 (30.2) | 982 (30.9) |
| Migration status (%) | Old migrants | 2290 (75.7) | 114 (71.7) | 2404 (75.5) |
|  | New migrants | 734 (24.3) | 45 (28.3) | 779 (24.5) |
| Administrative division (%) | Dhaka | 2034 (67.3) | 83 (52.2) | 2117 (66.5) |
|  | Khulna | 141 (4.7) | 12 (7.5) | 153 (4.8) |
|  | Rajshahi | 52 (1.7) | 4 (2.5) | 56 (1.8) |
|  | Other divisions | 797 (26.4) | 60 (37.7) | 857 (26.9) |
| Garbage disposal methods (%) | Disposed within premises | 156 (5.2) | 11 (6.9) | 167 (5.2) |
|  | Collected from home | 1050 (34.7) | 47 (29.6) | 1097 (34.5) |
|  | Disposed in bin outside | 384 (12.7) | 22 (13.8) | 406 (12.8) |
|  | Disposed in open spaces | 1434 (47.4) | 79 (49.7) | 1513 (47.5) |
| Ownership of dwelling (%) | Employer or other | 86 (2.8) | 7 (4.4) | 93 (2.9) |
|  | Owned | 506 (16.7) | 26 (16.4) | 532 (16.7) |
|  | Rented | 2430 (80.4) | 126 (79.2) | 2556 (80.3) |
|  | NA | 2 (0.1) | 0 (0.0) | 2 (0.1) |
| Ownership of land (%) | Government | 169 (5.6) | 12 (7.5) | 181 (5.7) |
|  | Landlord | 2402 (79.4) | 122 (76.7) | 2524 (79.3) |
|  | NGO | 27 (0.9) | 5 (3.1) | 32 (1.0) |
|  | Respondent or another resident | 424 (14.0) | 20 (12.6) | 444 (13.9) |
|  | NA | 2 (0.1) | 0 (0.0) | 2 (0.1) |
