# Supplementary material for: The effects of social determinants on children’s health outcomes in Bangladesh slums through an intersectionality lens: An application of multilevel analysis of individual heterogeneity and discriminatory accuracy (MAIHDA)
Source: PLOS Glob Public Health. 2023 Mar 8;3(3):e0001588. doi: 10.1371/journal.pgph.0001588 (PMC10022045; doi:10.1371/journal.pgph.0001588)
Supplement: S7 Table — (DOCX) [file pgph.0001588.s008.docx]

**S7 Table. Univariate analyses for acute respiratory infections (ARI).**

| Variable | Category | Coefficient | Standard Error | p-value |
| --- | --- | --- | --- | --- |
| Child sex | Female (Reference) |  |  |  |
|  | Male | 0.04 | 0.16 | 0.81 |
| Child age | 1 year and less (Reference) |  |  |  |
|  | 2 to 5 years | -0.40 | 0.16 | 0.01** |
| Mother age | <18 years (Reference) |  |  |  |
|  | 18 years and above | -0.61 | 0.38 | 0.11 |
| Mother religion | Islam (Reference) |  |  |  |
|  | Minority religion | -0.48 | 0.42 | 0.25 |
| Mother ever attended school | No (Reference) |  |  |  |
|  | Yes | 0.24 | 0.21 | 0.25 |
| Mother employment | No (Reference) |  |  |  |
|  | Yes | -0.43 | 0.22 | 0.04** |
| Mother marital status | Married (Reference) |  |  |  |
|  | Not married | -0.61 | 0.72 | 0.40 |
| Head age | 13 - 29 years (Reference) |  |  |  |
|  | 30 - 44 years | -0.68 | 0.19 | 0.00** |
|  | 45 years and above | 0.05 | 0.22 | 0.83 |
| Sex head | Female (Reference) |  |  |  |
|  | Male | -0.46 | 0.25 | 0.06 |
| Marital status head | Married (Reference) |  |  |  |
|  | Currently not married | 0.51 | 0.34 | 0.14 |
| Wealth index | Rich (Reference) |  |  |  |
|  | Middle | -0.48 | 0.30 | 0.11 |
|  | Poor | -0.28 | 0.25 | 0.26 |
| Housing condition | Multiple story (Reference) |  |  |  |
|  | Single story | -0.20 | 0.26 | 0.45 |
| Separate kitchen | No (Reference) |  |  |  |
|  | Yes | -0.06 | 0.17 | 0.74 |
| Cooking fuel used | Charcoal, dung cakes, etc. (Reference) |  |  |  |
|  | Kerosene or liquid gas | -0.20 | 0.64 | 0.76 |
|  | Natural gas | -0.59 | 0.38 | 0.12 |
|  | Wood fuel | -0.58 | 0.40 | 0.15 |
| Migration status | Old migrants (Reference) |  |  |  |
|  | New migrants | 0.21 | 0.18 | 0.25 |
| Administrative division | Dhaka (Reference) |  |  |  |
|  | Khulna | 0.73 | 0.32 | 0.02** |
|  | Rajshahi | 0.63 | 0.53 | 0.24 |
|  | Other divisions | 0.61 | 0.17 | 0.00** |
| Garbage disposal | Disposed within premises (Reference) |  |  |  |
|  | Collected from home | -0.45 | 0.35 | 0.19 |
|  | Disposed in bin outside | -0.20 | 0.38 | 0.60 |
|  | Disposed in open spaces | -0.24 | 0.33 | 0.46 |
| Ownership dwelling | Employer or other (Reference) |  |  |  |
|  | Owned | -0.45 | 0.44 | 0.30 |
|  | Rented | -0.45 | 0.40 | 0.27 |
| Ownership land | Government (Reference) |  |  |  |
|  | Landlord | -0.33 | 0.31 | 0.29 |
|  | NGO | 0.96 | 0.57 | 0.09 |
|  | Respondent or another resident | -0.40 | 0.38 | 0.29 |

** Statistically Significant at 5% level of significance.
