# Supplementary material for: The effects of social determinants on children’s health outcomes in Bangladesh slums through an intersectionality lens: An application of multilevel analysis of individual heterogeneity and discriminatory accuracy (MAIHDA)
Source: PLOS Glob Public Health. 2023 Mar 8;3(3):e0001588. doi: 10.1371/journal.pgph.0001588 (PMC10022045; doi:10.1371/journal.pgph.0001588)
Supplement: S8 Table — (DOCX) [file pgph.0001588.s009.docx]

**S8 Table. Calculations of intersectional strata for each of three outcomes**

| Outcome | Statistically significant variable in univariate analysis (No. of category) | Supplementary information table | Interaction among categories (combinations) | Initial number of intersectional strata | Number of strata with zero observation | Final no. of intersectional strata |
| --- | --- | --- | --- | --- | --- | --- |
| 1 | 2 | 3 | 4 | 5=4 | 6 | 7=5-6 |
| Cough | - Child age (2) - Division (4) - Age of household head (3) - Garbage disposal (4) - Mothers’ employment (2) | S5 Table | 2*4*3*4*2 | 192 | 45 | 147 |
| Fever | - Child age (2) - Division (4) - Age of household head (3) - Garbage disposal (4) - Mothers’ age (2) - Mother’ employment (2) - Mother ever attended school (2) - Separate kitchen (2) | S6 Table | 2*4*3*2*4*2*2*2 | 1536 | 1095 | 441 |
| ARI | - Child age (2) - Division (4) - Age of household head (3) - Wealth index (3) - Cooking fuel used (4) - Mothers’ employment (2) | S7 Table | 2*4*3*3*4*2 | 576 | 329 | 247 |
