# Supplementary material for: The effects of social determinants on children’s health outcomes in Bangladesh slums through an intersectionality lens: An application of multilevel analysis of individual heterogeneity and discriminatory accuracy (MAIHDA)
Source: PLOS Glob Public Health. 2023 Mar 8;3(3):e0001588. doi: 10.1371/journal.pgph.0001588 (PMC10022045; doi:10.1371/journal.pgph.0001588)
Supplement: Attachment — Submitted filename: Response to Reviewers.docx [file pgph.0001588.s010.docx]

Journal Requirements:

1. Please amend your detailed online Financial Disclosure statement. This is published with the article. It must therefore be completed in full sentences and contain the exact wording you wish to be published.

Please state what role the funders took in the study. If the funders had no role in your study, please state: “The funders had no role in study design, data collection and analysis, decision to publish, or preparation of the manuscript.”

- **Online Financial Disclosure statement: “***The GCRF Accountability for Informal Urban Equity Hub (‘ARISE’) is a UKRI Collective Fund award with award reference ES/S00811X/1. EK, AHL and LG are also funded by funded by the Medical Research Council (MC_UU_00022/2) and the Scottish Government Chief Scientist Office (SPHSU17). The funders had no role in study design, data collection and analysis, decision to publish, or preparation of the manuscript”*

Reviewers' comments:

Reviewer's Responses to Questions

**Comments to the Author**

1. Does this manuscript meet PLOS Global Public Health’s [publication criteria](https://journals.plos.org/globalpublichealth/s/criteria-for-publication)? Is the manuscript technically sound, and do the data support the conclusions? The manuscript must describe methodologically and ethically rigorous research with conclusions that are appropriately drawn based on the data presented.

Reviewer #1: Yes

Reviewer #2: Partly

2. Has the statistical analysis been performed appropriately and rigorously?

Reviewer #1: I don't know

Reviewer #2: No

3. Have the authors made all data underlying the findings in their manuscript fully available (please refer to the Data Availability Statement at the start of the manuscript PDF file)?

The [PLOS Data policy](https://journals.plos.org/globalpublichealth/s/data-availability) requires authors to make all data underlying the findings described in their manuscript fully available without restriction, with rare exception. The data should be provided as part of the manuscript or its supporting information, or deposited to a public repository. For example, in addition to summary statistics, the data points behind means, medians and variance measures should be available. If there are restrictions on publicly sharing data—e.g. participant privacy or use of data from a third party—those must be specified.

Reviewer #1: Yes

Reviewer #2: No

4. Is the manuscript presented in an intelligible fashion and written in standard English?

PLOS Global Public Health does not copyedit accepted manuscripts, so the language in submitted articles must be clear, correct, and unambiguous. Any typographical or grammatical errors should be corrected at revision, so please note any specific errors here.

Reviewer #1: Yes

Reviewer #2: Yes

5. Review Comments to the Author

Please use the space provided to explain your answers to the questions above. You may also include additional comments for the author, including concerns about dual publication, research ethics, or publication ethics. (Please upload your review as an attachment if it exceeds 20,000 characters)

**Reviewer #1:**

Overall, the authors have worked diligently to conduct a statistical analysis using MAIHDA to evaluate the effects of SDoH on child health outcomes in Bangladeshi slums. I was not familiar with this statistical methodology prior to reviewing this work, which may contribute to my relative lack of understanding of its application here. While I believe this work could be impactful, this manuscript needs revision in a few aspects before it is ready for publication.

- *Thank you for reviewing the manuscript and we greatly appreciate your valuable time and effort. We have addressed all comments and suggestions in the revised manuscript.*

1. It is unclear why cough is separate from ARI as a cause of mortality. Can the authors say more about how they chose these three health outcomes to evaluate? Why was diarrhea not included? The paragraph in the introduction where the causes of child mortality is introduced is a bit hard to follow, as it begins by stating that fever, cough, and ARI are common causes of child mortality but then goes on to discuss prevalence of ARI and then return to mortality in the middle of the paragraph, then back to ARI hospitalization rate. Would rework the introduction to be more direct and clearly state why these three health outcomes were selected.

- *Thank you for pointing out thus. We wish we could include “diarrhoea” as one the health outcomes. However, we could not include “diarrhoea” due to lack of data in* ***“Bangladesh Urban Health Survey (UHS) 2013”*** *.*
- *You are right Cough and ARI are not separated as a cause of mortality and in fact ARI is defined in the survey as “**cough accompanied by short, rapid, or difficult breathing which is chest related. It is considered as proxy pneumonia” So, we have revised the statement in the revised manuscript.*

2. It is not clear to me how the intersectional strata are defined. It is entirely possible that this is due to my personal unfamiliarity with this methodology, but the methods section does not clearly explain this to the average researcher.

- *We have added one more Supplementary Information (S8 Table ) to show the calculations of intersectional strata by each of three outcomes as follows. This may help readers better understand the definition of intersectional strata.*

**S8 Table. Calculations of intersectional strata for each of three outcomes**

| Outcomes | Statistically significant variable in univariate analysis (No of category) | Supplementary information | Interaction among categories (combinations) | Initial number of intersectional strata | Number of strata with zero observation | Final no. of intersectional strata |
| --- | --- | --- | --- | --- | --- | --- |
| 1 | 2 | 3 | 4 | 5=4 | 6 | 7=5-6 |
| Cough | - Child age (2) - Division (4) - Age of household head (3) - Garbage disposal (4) - Mothers’ employment (2) | S5 Table | 2*4*3*4*2 | 192 | 45 | 147 |
| Fever | - Child age (2) - Division (4) - Age of household head (3) - Garbage disposal (4) - Mothers’ age (2) - Mother’ employment (2) - Mother ever attended school (2) - Separate kitchen (2) | S6 Table | 2*4*3*2*4*2*2*2 | 1536 | 1095 | 441 |
| ARI | - Child age (2) - Division (4) - Age of household head (3) - Wealth index (3) - Cooking fuel used (4) - Mothers’ employment (2) | S7 Table | 2*4*3*3*4*2 | 576 | 329 | 247 |

3. The statistical analysis reported varied factors for each of the three health outcomes. It is unclear why certain social determinant factors were reported for cough vs fever vs ARI – it makes it difficult to understand the impact of SDoH on health outcomes in aggregate if it not clearly stated which determinant factors are included in each analysis.

- *“We have selected social determinants of health (SDoH) based on their statistical significance with respective outcomes in the univariate analyses”. In addition, we have mentioned that this univariate approach has some limitations. We have added texts in the Measures sub-section of Methods section of the manuscript. Please refer to Supplementary Information (S5, S6 and S7 Tables) for choosing respective SDoH factors by outcomes as follows.*

| Outcomes | Statistically significant SDoH | Supplementary information |
| --- | --- | --- |
| Cough | - Child age - Division - Age of household head - Garbage disposal - Mothers’ employment | S5 Table |
| Fever | - Child age - Division - Age of household head - Garbage disposal - Mothers’ age - Mother’ employment - Mother ever attended school - Separate kitchen | S6 Table |
| ARI | - Child age - Division - Age of household head - Wealth index - Cooking fuel used - Mothers’ employment | S7 Table |

4. The conclusions drawn in the discussion are not necessarily supported by this evidence as there is moderate and disparate effects of SDoH that can be drawn for each of the three health outcomes. Also, it is unclear how these analyses provide additional meaningful information beyond what has been published about the association of SDoH and health outcomes in pediatric populations, as the authors reference extensively in the introduction.

- *We have added these texts inn the revised manuscript “While previous studies mostly investigated the association between social determinants and health outcomes, this study has extended those analyses by evaluating the association of collective SDoH and health outcomes for children under five years old (U5y) via intersectional approach. Intersectional approach helps understand health inequalities based on multiple dimensions of identity (social strata). This helps tackle health inequalities universally across all social strata created in scale proportionate to the level of disadvantage and not only targeting a specific group. Therefore, based on the analyses we have found that some children in certain intersectional strata are more vulnerable compared to others and therefore need a greater level of interventions compared to other groups despite all coming from already marginalized population of urban slum dwellers. For example, older children (2 to 5 years) with younger household head (13 to 29 years) household having better waste disposal system (bin outside) and unemployed mother and living in Dhaka division are more vulnerable to having a cough (stratum 147 of in Fig 2). Similarly, older children (2 to 5 years) with older household head (30 to 44 years) and from households having better waste management system (collected from home) and separate kitchen, employed mother aged 18 years and above living in Dhaka division are more vulnerable to having a fever (stratum 441 in Fig 2). Older children (2 to 5 years) from households of middle wealth quintile with older household head (30 to 44 years) and unemployed mother, household using natural gas and living in Dhaka division are more vulnerable to having an ARI (stratum 247 in Fig 3).”*

- *We have specified the recommendations based on our findings. For example, based on the value of Proportional Change in Variance (PCV) we have identified intersectional effects exists based on the social determinants we have incorporated in the model. This is crucial because we have identified which children based on the combination of social determinants (i.e., groups) are more prone to worse health outcomes. In turn, this will help policy makers develop interventions which are proportionate to the level of vulnerability.*

5. The authors reference risks of each health outcome relative to each division of slums. Perhaps it would be more illustrative to use a map to highlight the significant risks present in each division, to allow the reader to create a relational understanding of the risks in Dhaka slums vs others around the country (which is referenced in the conclusions and policy implications).

- *As suggested, we have added a map showing all divisions of Bangladesh to illustrate better when describing risks of each health outcome. Additionally, other divisions include other divisions including Barisal, Chittagong, Rangpur or Sylhet (Fig.1). We changed the numbering of subsequent figures accordingly.*

- ***Fig 4. Children under five years old (U5y) at risk of cough, fever, and ARI across slums in seven divisions of Bangladesh***
- ***This is the Fig 4 legend***
- *In this study we combined Barisal, Chittagong, Rangpur or Sylhet divisions as “other divisions”. U5y living in slums in other divisions were at higher risk of having cough, fever and ARI than those living in Dhaka. Division. Similarly, U5y living in Rajshahi and Khulna divisions are more likely to have cough and ARI respectively compared to those living slums in in Dhaka Division.*

6. Try to avoid language that could be viewed as insulting or pejorative. For example, would not use the phrase “slum people”.

- *We have removed the term “slum people” and replaced it with “people living in slums” in the text*

7. At times, the word choice and grammar are confusing and unclear, which contributed to the difficulty I had in understanding the text. I would recommend reviewing for grammar, typos, and clarity.

- *We have reviewed the manuscript for grammar, typos, and clarity and revised accordingly. For instance, we have added “quintiles of” in the text of revised manuscript. We have added these texts “However, if the VPC in Model 2 is not equal to zero, it suggests presence of interactional effects”. We have added "1” in the text which was missing. We have corrected the value. It will be 0.5 instead of 0.05, We have corrected by adding “statistically significant at 5% level of significance” in the Table 1 legend. We have corrected by adding “strata variance in outcome” in the Table 1. We added “people living in slums” instead of “slum people”.*

**Reviewer #2:**

The study aims to examine the effects of social determinants of children’s health outcomes in slums in Bangladesh. Association between individual social determinants and health outcomes have previously been analyzed and established; arguably, the authors method adds value by looking at the same question from an intersectional approach. I enjoyed reading the piece, and would like to highlight a few other areas where the manuscript can be strengthened with regard to its scientific merit.

- *Thank you for reviewing the manuscript and we greatly appreciate. We have addressed all comments and suggestions in the revised manuscript.*

1) Avoid causal language throughout the manuscript. There shouldn’t really be any reference to “effects” since this is an associational study.

- *Thank you for bringing this to our attention. We are aware that this not an experimental study and we are not looking for causality or impact of intervention on outcomes as well. However, for the sake of convention we have used “intersectional effects” and “main effects” to show the coefficients of Model 1 and Model 2 respectively. To avoid any confusion, we have used “intersectional effects estimates” and “main effects estimates” throughout the revised manuscript.*

2) There needs to be a much clearer discussion—in the introduction—of what the authors mean by intersectional. The authors never fully explain it anywhere.

- We have added these texts with reference (Heard et al., 2020) in the introduction section of the revised manuscript. “The concept of intersectionality describes the phenomenon in which social determinants such as gender, race, ethnicity, sexual orientation, religion, disability, class and other forms of identity “intersect” to create unique dynamics and effects (Heard et al., 2020). When these identities do not work independently but interact, they lead to aggravate social inequities. We have used the intersectionality concept to identify group level effects (termed as “intersectional effects” in this paper) and individual levels effects (termed as “main effects” in this paper) in under-five health outcomes (Merlo, 2018).”

3) Related to (2), please explain the advantage of using MAIHDA compared to using a general approach (regression with interaction terms for various combinations of the independent variables). The distinction between the two—and the justification for using MAIHDA—is important because the authors have categorized several variables that could have been used as continuous variables—thus retaining and utilizing the variation in the variable—if they had not used MAIHDA. Also, my understanding is that it is relatively straightforward to obtain marginal effect of interaction terms in regression models (essentially getting at the intersectional aspect, from what I understand), so I am not clear what MAIHDA buys us here.

- Regarding advantage of MAIHDA over conventional model, we have added a paragraph in Statistical methods sub-section of Methods section of the revised manuscript as follows “MAIHDA approach has advantages over conventional fixed-effect approaches such as precision‐weighted estimates, model parsimony, ease of interpretation, and reliable estimates for strata with small sample sizes (Evans et al., 2018; Merlo, 2018) as stratum with few observations can pool information from others via multilevel modelling to obtain better inferences (Jaehn et al., 2020). Additionally, MAIHDA allows for the inductive analysis of numerous stratum-specific interactions and it avoids pre-specifying specific interactions or intersections to explore (Evans et al., 2018; Li & Spini, 2022; Merlo, 2018; Wemrell et al., 2021). Most importantly, MAIHDA estimates the measures of discriminatory accuracy (DA) together with traditional comparison of population averages leading to enable more comprehensively informed decisions about suitable public health action to address an issue under study. MAIHDA provides a feasible way of measuring multiple intersections and analyzing groups of small size in an intersectionality perspective. MAIDHA also provides a parsimonious way of analyzing high dimensional interactions which is not possible with single level models (Jaehn et al., 2020; Merlo, 2018).”
- In conventional models it is not possible to compare all interactions together. That is, in conventional models : Inclusion of interaction terms encourages to only study intersectionality from the perspective of marginalization. This denies the chance to investigate those groups enjoying multiple privileges or mix of privilege and marginalization. Comparing the multiply marginalized to the multiply privileged runs the risk of reinforcing the notion that the dominant, privileged groups are the yardstick against which other groups need to be compared with.
- Reference

Evans, C. R., Williams, D. R., Onnela, J.-P., & Subramanian, S. V. (2018). A multilevel approach to modeling health inequalities at the intersection of multiple social identities. *Social Science & Medicine (1982)*, *203*, 64–73. https://doi.org/10.1016/j.socscimed.2017.11.011

Heard, E., Fitzgerald, L., Wigginton, B., & Mutch, A. (2020). Applying intersectionality theory in health promotion research and practice. *Health Promotion International*, *35*(4), 866–876. https://doi.org/10.1093/heapro/daz080

Holman, D., Salway, S., & Bell, A. (2020). Mapping intersectional inequalities in biomarkers of healthy ageing and chronic disease in older English adults. *Scientific Reports*, *10*(1), 13522. https://doi.org/10.1038/s41598-020-69934-8

Jaehn, P., Mena, E., Merz, S., Hoffmann, R., Gößwald, A., Rommel, A., Holmberg, C., & ADVANCE GENDER study group. (2020). Non-response in a national health survey in Germany: An intersectionality-informed multilevel analysis of individual heterogeneity and discriminatory accuracy. *PloS One*, *15*(8), e0237349. https://doi.org/10.1371/journal.pone.0237349

Li, Y., & Spini, D. (2022). Intersectional social identities and loneliness: Evidence from a municipality in Switzerland. *Journal of Community Psychology*, *50*(8), 3560–3573. https://doi.org/10.1002/jcop.22855

Merlo, J. (2018). Multilevel analysis of individual heterogeneity and discriminatory accuracy (MAIHDA) within an intersectional framework. *Social Science & Medicine (1982)*, *203*, 74–80. https://doi.org/10.1016/j.socscimed.2017.12.026

Wemrell, M., Karlsson, N., Perez Vicente, R., & Merlo, J. (2021). An intersectional analysis providing more precise information on inequities in self-rated health. *International Journal for Equity in Health*, *20*(1), 54. https://doi.org/10.1186/s12939-020-01368-0

4) Also related, instead of picking variables for strata based on statistical significance in univariate analysis, why not base it on existing literature and on authors’ understanding of the context? Or, based on variables over which policymakers have some control (e.g., employment, education, housing conditions)?

- *Initially, the explanatory variables used in the study were selected based on existing literature on factors that influence health outcomes for U5y as well as informed by the framework developed by Commission for Social Determinants of Health (CSDH). However, for strata formation we included all variables that showed statistical significance (a p-value less than 0.05) on univariate analysis.*

5) On page 8, the authors need to explain the method more clearly. S1 File is useful. I wish the part in the main text was written in more plain language.

- *Thank you for pointing out this. We have described methodology in detail in our protocol paper and we refer to our published paper (Kibuchi et al., 2022) in the manuscript.*
- Reference

Kibuchi E, Barua P, Chumo I, Teixeira de Siqueira Filha N, Phillips-Howard P, Mithu MIH, et al. Effects of social determinants on children’s health in informal settlements in Bangladesh and Kenya through an intersectionality lens: a study protocol. BMJ Open [Internet]. 2022 Jun [cited 2022 Jun 12];12(6):e056494. Available from: https://bmjopen.bmj.com/lookup/doi/10.1136/bmjopen-2021-056494

6) In the methods (and in the method appendix), there is reference to “assuming no relevant variables are omitted suggesting presence of interaction effect”. This seems like a big assumption. How do we know that it holds

- *Thank you for pointing out this. This is MAIHDA assumptions for analysis. However, in the limitations sub-section of the manuscript we have mentioned this limitation “it was not possible to include all variables because our analyses included main effects that only showed statistical significance (a p-value less than 0.05) on univariate analysis”*

7) Stylistically, please consider reorganizing the results section. Results in 3 outcomes are explained separately. Tables 1, 2 and 3 can be combined into one table, and the results can be explained more coherently—focusing on the key points.

- *We have combined Table 1, 2 and 3 and explained the key points more coherently. See Table 1 below*
- ***Table 1: Main effects coefficient, strata variance, area under the curve, variance partition coefficient (VPC) and proportional change in variance (PCV) for cough (Model 1 and 2), fever (Model 1 and 2), and ARI (Model 1 and 2)***

|  |  | **Cough** | | | | **Fever** | | | | **ARI** | | | |
| --- | --- | --- | --- | --- | --- | --- | --- | --- | --- | --- | --- | --- | --- |
|  |  | **Model 1** | | **Model 2** | | **Model 1** | | **Model 2** | | **Model 1** | | **Model 2** | |
|  | Category (Reference) | Odds Ratio | 95% CI^a^ | Odds Ratio | 95% CI^a^ | Odds Ratio | 95% CI^a^ | Odds Ratio | 95% CI^a^ | Odds Ratio | 95% CI^a^ | Odds Ratio | 95% CI^a^ |
| Intercept |  | 0.24** | (0.21, 0.28) | 0.47** | (0.31, 0.71) | 0.39** | (0.36, 0.43) | 0.75 | (0.42,1.31) | 0.050** | (0.04, 0.06) | 0.10** | (0.03, 0.27) |
| Child age | Age 1 year and less (Reference) |  |  | 1.00 |  |  |  | 1.00 |  |  |  | 1.00 |  |
|  | Age 2 to 5 years old |  |  | 0.76** | (0.64, 0.96) |  |  | 0.89 | (0.74,1.07) |  |  | 0.78 | (0.54, 1.12) |
| Division | Dhaka (Reference) |  |  | 1.00 |  |  |  |  |  |  |  | 1.00 |  |
|  | Khulna |  |  | 1.41 | (0.91, 2.15) |  |  | 1.10 | (0.74,1.60) |  |  | 2.46** | (1.14, 5.17) |
|  | Rajshahi |  |  | 1.94** | (1.00, 3.48) |  |  | 1.52 | (0.84,2.69) |  |  | 1.66 | (0.45, 4.98) |
|  | Other divisions |  |  | 1.76** | (1.34, 2.16) |  |  | 1.50** | (1.22,1.84) |  |  | 2.06** | (1.39, 3.07) |
| Age of the household head | Age 13-29 years old (Reference) |  |  | 1.00 |  |  |  | 1.00 |  |  |  | 1.00 |  |
|  | Age 30 to 44 years old |  |  | 0.62** | (0.54, 0.87) |  |  | 0.66** | (0.54,0.80) |  |  | 0.48** | (0.31, 0.73) |
|  | Age 45 years and above |  |  | 0.81 | (0.66, 1.18) |  |  | 0.81 | (0.63,1.05) |  |  | 0.88 | (0.52, 1.43) |
| Garbage Disposal | Disposed within premises (Reference) |  |  | 1.00 |  |  |  | 1.00 |  |  |  |  |  |
|  | Collected from home |  |  | 0.55** | (0.39, 0.90) |  |  | 0.65** | (0.45,0.95) |  |  |  |  |
|  | Disposed in bin outside |  |  | 0.54** | (0.36, 0.89) |  |  | 0.63** | (0.42,0.95) |  |  |  |  |
|  | Disposed in open spaces |  |  | 0.51** | (0.38,0.84) |  |  | 0.74 | (0.52,1.06) |  |  |  |  |
| Mothers’ employment | No (Reference) |  |  | 1.00 |  |  |  | 1.00 |  |  |  | 1.00 |  |
|  | Yes |  |  | 0.75** | (0.60, 0.98) |  |  | 1.15 | (0.94,1.42) |  |  | 0.67 | (0.42, 1.06) |
| Mothers’ age | Age below 18 years old (Reference) |  |  |  |  |  |  | 1.00 |  |  |  |  |  |
|  | Age 18 and above |  |  |  |  |  |  | 0.72 | (0.46,1.14) |  |  |  |  |
| Mother ever attended school | No (Reference) |  |  |  |  |  |  | 1.00 |  |  |  |  |  |
|  | Yes |  |  |  |  |  |  | 0.84 | (0.68,1.03) |  |  |  |  |
| Separate kitchen | No (Reference) |  |  |  |  |  |  | 1.00 |  |  |  |  |  |
|  | Yes |  |  |  |  |  |  | 1.15 | (0.96,1.37) |  |  |  |  |
| Cooking fuel used | Charcoal, dung cakes, etc. (Reference) |  |  |  |  |  |  |  |  |  |  | 1.00 |  |
|  | Kerosene or liquid gas |  |  |  |  |  |  |  |  |  |  | 0.82 | (0.19, 3.06) |
|  | Natural gas |  |  |  |  |  |  |  |  |  |  | 0.67 | (0.30, 1.63) |
|  | Wood fuel |  |  |  |  |  |  |  |  |  |  | 0.48 | (0.21, 1.19) |
| Wealth index | Reference (Rich) |  |  |  |  |  |  |  |  |  |  | 1.00 |  |
|  | Middle |  |  |  |  |  |  |  |  |  |  | 0.74 | (0.39, 1.40) |
|  | Poor |  |  |  |  |  |  |  |  |  |  | 0.93 | (0.54, 1.66) |
| Strata variance in outcome |  | 0.18 |  | 0.05 |  | 0.13 |  | 0.02 |  | 0.14 |  | 0.04 |  |
| Strata N |  | 147 |  | 147 |  | 441 |  | 441 |  | 247 |  | 247 |  |
| Individual |  | 3,173 |  | 3,173 |  | 3,183 |  | 3,183 |  | 3,183 |  | 3,183 |  |
| AUC-ROC^b^ |  | 64.8% |  | 61.9% |  | 68% |  | 61.89% |  | 72.93% |  | 66.36% |  |
| VPC^c^ |  | 5.25% |  | 0.77% |  | 3.91% |  | 0.87% |  | 4.24% |  | 1.32% |  |
| PCV^d^ |  |  |  | 85.90% |  |  |  | 78.42% |  |  |  | 69.77% |  |

- ***This is the Table 1 legend.***

^a^95% CI: 95% Credible Interval with lower bound, upper bound in parentheses.

^b^AUC-ROC: Area Under the Receiver Operating Characteristic Curve.

^c^VPC: Variance Partition Coefficient.

^d^PCV: Proportional Change in Variance.

** Statistically Significant at 5% level of significance.

8) Minor: based on the findings of the study, what do we now know that we did not from so many previous studies that have been conducted on this topic (at least from the policy perspective in Bangladesh)? Some recommendations are made in the Conclusion, but they seem too general to be useful.

- *While previous studies mostly investigated the association between social determinants and health outcomes this study has extend those analyses by evaluating the association of collective SDoH and health outcomes for children under five years old (U5y) via intersectional approach. We have specified the recommendations based on our findings. We find low discriminatory accuracy of the intersectional strata suggesting universal interventions rather than interventions exclusively focused on strata with a higher average risk of ARI, cough or fever.*

Other minor points:
• Line 132 – the authors likely mean the quintiles of wealth index (of from S1 – categories of wealth index), not wealth index, as the method requires categorical variables

- *Done: We have added “*quintiles of” in the text of revised manuscript.

• Line 158-159 – incomplete sentence

- *We have added these texts “However, if the VPC in Model 2 is not equal to zero, it suggests presence of interactional effects”*

• Line 164 – “1” missing

- *We have added "1” in the text*

• Line 165 – Is it 0.5 to 1 or 0.05 to 1? The previous sentence says 0.5.

- *We have corrected the value. It will be 0.5 instead of 0.05*

• Line 186 – I’d put the link to the 2013 Urban Health Survey page, not the general link to UNC’s dataverse.

- *Thank you for your suggestion. We have revised by using the direct website link* [*https://dataverse.unc.edu/dataset.xhtml?persistentId=doi:10.15139/S3/12274*](https://dataverse.unc.edu/dataset.xhtml?persistentId=doi:10.15139/S3/12274) *for accessing the dataset named “Bangladesh Urban Health Survey (UHS) 2013” and removed UNC’s general dataverse link (*[*https://dataverse.unc.edu*](https://dataverse.unc.edu)*).*

• Line 197 – Explain how you created 147 strata for cough. There seem to be 9 coefficients that are statistically significant. How do we go from those 9 coefficients to 147 strata? Similar explanation can work for how 441 and 247 strata were created for fever and ARI.

- *We have added one more table in the Supplementary Information main text (S8 Table ) to show the calculations of intersectional strata by each of three outcomes as follows.*
- ***S8 Table. Calculations of intersectional strata for each of three outcomes***

| Outcomes | Statistically significant variable in univariate analysis (No of category) | Supplementary information | Interaction among categories (combinations) | Initial number of intersectional strata | Number of strata with zero observation | Final no. of intersectional strata |
| --- | --- | --- | --- | --- | --- | --- |
| 1 | 2 | 3 | 4 | 5=4 | 6 | 7=5-6 |
| Cough | - Child age (2) - Division (4) - Age of household head (3) - Garbage disposal (4) - Mothers’ employment (2) | S5 Table | 2*4*3*4*2 | 192 | 45 | 147 |
| Fever | - Child age (2) - Division (4) - Age of household head (3) - Garbage disposal (4) - Mothers’ age (2) - Mother’ employment (2) - Mother ever attended school (2) - Separate kitchen (2) | S6 Table | 2*4*3*2*4*2*2*2 | 1536 | 1095 | 441 |
| ARI | - Child age (2) - Division (4) - Age of household head (3) - Wealth index (3) - Cooking fuel used (4) - Mothers’ employment (2) | S7 Table | 2*4*3*3*4*2 | 576 | 329 | 247 |

• Table 1 – Significant at what level? Also, the authors likely mean *statistically* significant.

- *We have corrected by adding “statistically significant at 5% level of significance” in the Table 1 legend*

• Table 1 – Strata variance in what? Is this for outcome?

- *We have corrected by adding “strata variance in outcome” in the Table 1*

• In S3/S4 Table, mothers with no education seem to be in both ‘Mother Ever Attended School’ and ‘Mother’s Highest Education’. The first one seems redundant.

- *We have removed one variable ‘Mother’s Highest Education’ from all supplementary tables (S1 – S7) and from the main text as well to avoid redundancy. We kept ‘Mother Ever Attended School’ due to statistically significant relationship with outcome (fever).*

6. PLOS authors have the option to publish the peer review history of their article ([what does this mean?](https://journals.plos.org/globalpublichealth/s/editorial-and-peer-review-process#loc-peer-review-history)). If published, this will include your full peer review and any attached files.


**Do you want your identity to be public for this peer review?** If you choose “no”, your identity will remain anonymous but your review may still be made public.


For information about this choice, including consent withdrawal, please see our [Privacy Policy](https://www.plos.org/privacy-policy).

Reviewer #1: No

Reviewer #2: No
