# Supplementary material for: The effects of social determinants on children’s health outcomes in Bangladesh slums through an intersectionality lens: An application of multilevel analysis of individual heterogeneity and discriminatory accuracy (MAIHDA)
Source: PLOS Glob Public Health. 2023 Mar 8;3(3):e0001588. doi: 10.1371/journal.pgph.0001588 (PMC10022045; doi:10.1371/journal.pgph.0001588)
Supplement: Attachment — Submitted filename: Response to Reviewers.docx [file pgph.0001588.s011.docx]

**Journal Requirements:**

1. Please review your reference list to ensure that it is complete and correct. If you have cited papers that have been retracted, please include the rationale for doing so in the manuscript text, or remove these references and replace them with relevant current references. Any changes to the reference list should be mentioned in the rebuttal letter that accompanies your revised manuscript. If you need to cite a retracted article, indicate the article’s retracted status in the References list and also include a citation and full reference for the retraction notice.

- ***We have reviewed the references which are complete and correct. We did not use any retracted article in the manuscript. Please find the reference list before revision (68 references) and after revision (66 references). We removed duplicates .***

***After revision***

| **Ref #** | **Reference style: Vancouver** |
| --- | --- |
| 1 | Hossain B. Slum of Bangladesh: An Overview [Internet]. 2020 [cited 2021 Dec 6]. Available from: https://www.researchgate.net/publication/339569720_Slum_of_Bangladesh_An_Overview |
| 2 | Islam N, Mahbub A, Islam N. Urban slums of Bangladesh [Internet]. The Daily Star. 2009 [cited 2021 Dec 4]. Available from: https://www.thedailystar.net/news-detail-93293 |
| 3 | Bangladesh Bureau of Statistics. Preliminary Report on the Census of Slum Areas and Floating Population 2014. Dhaka, Bangladesh: Ministry of Planning Government of the People’s Republic of Bangladesh; 2015 p. 1–45. |
| 4 | NIPORT, icddr,b, MEASURE Evaluation. Bangladesh Urban Health Survey 2013 Final Report. Chapel Hill, USA; 2015 p. 1–219. |
| 5 | Latif MB, Irin A, Ferdaus J. Socio-economic and health status of slum dwellers of the Kalyanpur slum in Dhaka city. Bangladesh J Sci Res. 2016;29(1):73–83. |
| 6 | Bangladesh Bureau of Statistics, UNICEF Bangladesh. Child well-being survey in urban areas of Bangladesh 2016: Key Results. Dhaka, Bangladesh; 2016 p. 1–23. |
| 7 | Islam MM, Debnath J, Rakhi NN, Al-Jubayer A. Epidemiologic pattern of respiratory diseases among hospitalized pediatric patients in Khulna, Bangladesh. Int J Sci Rep. 2021;7(6):306. |
| 8 | Rahman A, Hossain MdM. Prevalence and determinants of fever, ARI and diarrhea among children aged 6–59 months in Bangladesh. BMC Pediatr. 2022;22(1):117. |
| 9 | Mberu BU, Haregu TN, Kyobutungi C, Ezeh AC. Health and health-related indicators in slum, rural, and urban communities: a comparative analysis. Global Health Action. 2016;9(1):33163. |
| 10 | Zanuzdana A, Khan MMH, Brinkel JK, Kraemer A. Factors associated with high prevalence of coughs, colds and fever among urban slum dwellers in Dhaka, Bangladesh. SE Asia J Pub Health. 2014;3(2):58–65. |
| 11 | Sayem AM, Nury ATMdS, Hossain MdD. Achieving the Millennium Development Goal for Under-five Mortality in Bangladesh: Current Status and Lessons for Issues and Challenges for Further Improvements. J Health Popul Nutr. 2011 Apr;29(2):92–102. |
| 12 | Dewey KG, Mayers DR. Early child growth: how do nutrition and infection interact?: How do nutrition and infection interact? Maternal & Child Nutrition. 2011 Oct;7:129–42. |
| 13 | Biermann O, Mwoka M, Ettman CK, Abdalla SM, Shawky S, Ambuko J, et al. Data, Social Determinants, and Better Decision-making for Health: the 3-D Commission. J Urban Health. 2021;98(S1):4–14. |
| 14 | Kundu S, Kundu S, Banna MHA, Ahinkorah BO, Seidu AA, Okyere J. Prevalence of and factors associated with childhood diarrhoeal disease and acute respiratory infection in Bangladesh: an analysis of a nationwide cross-sectional survey. BMJ Open. 2022 Apr 6;12(4):1–13. |
| 15 | Ullah MB, Mridha MK, Arnold CD, Matias SL, Khan MSA, Siddiqui Z, et al. Factors associated with diarrhea and acute respiratory infection in children under two years of age in rural Bangladesh. BMC Pediatr. 2019;19(1):386. |
| 16 | Solar O, Irwin A. A conceptual framework for action on the social determinants of health. Social Determinants of Health Discussion Paper 2 (Policy and Practice). Geneva, Switzerland: World Health Organization; 2010 p. 1–79. (Social Determinants of Health, 2). |
| 17 | Ahmed I. Factors in building resilience in urban slums of Dhaka, Bangladesh. Procedia Economics and Finance. 2014;18:745–53. |
| 18 | Perry H, Weierbach R, Hossain I, Islam R. Childhood immunization coverage in zone 3 of Dhaka City: the challenge of reaching impoverished households in urban Bangladesh. Bull World Health Organ. 1998;76(6):565–73. |
| 19 | Tarlov AR. Social determinants of health: The sociobiological translation. In: Blane D, Brunner E, Wilkinson R, editors. Health and Social Organization: Towards a Health Policy for the 21st Century. 1st ed. London: Routledge; 1996. p. 87–109. |
| 20 | Dovey K, King R. Forms of Informality: Morphology and Visibility of Informal Settlements. Built Environment (1978-). 2011;37(1):11–29. |
| 21 | WHO Kobe Centre. A billion voices: listening and responding to the health needs of slum dwellers and informal settlers in new urban settings. Kobe: Japan: World Health Organization (WHO); 2005 p. 1–50. |
| 22 | Heard E, Fitzgerald L, Wigginton B, Mutch A. Applying intersectionality theory in health promotion research and practice. Health Promotion International. 2020 Aug 1;35(4):866–76. |
| 23 | Merlo J. Multilevel analysis of individual heterogeneity and discriminatory accuracy (MAIHDA) within an intersectional framework. Soc Sci Med. 2018;203:74–80. |
| 24 | Zahan N, Sayed A, Mamun M, Sabiruzzaman N, Islam, Hossain A, et al. Factors Associated with Acute Respiratory Infections among Under-Five Children in Bangladesh. International Journal of Statistical Sciences. 2016 Jan 1;15(Special Issue):85–100. |
| 25 | Sultana M, Sarker AR, Sheikh N, Akram R, Ali N, Mahumud RA, et al. Prevalence, determinants and health care-seeking behavior of childhood acute respiratory tract infections in Bangladesh. PLoS ONE. 2019;14(1):1–18. |
| 26 | Mostafa I, Naila NN, Mahfuz M, Roy M, Faruque ASG, Ahmed T. Children living in the slums of Bangladesh face risks from unsafe food and water and stunted growth is common. Acta Paediatr. 2018 Jul;107(7):1230–9. |
| 27 | Yaya S, Bishwajit G. Burden of Acute Respiratory Infections Among Under-Five Children in Relation to Household Wealth and Socioeconomic Status in Bangladesh. TropicalMed. 2019 Feb 12;4(1):36. |
| 28 | Rahman MM, Shahidullah M. Risk factors for acute respiratory infections among the slum infants of Dhaka city. Bangladesh Med Res Counc Bull. 2001 Aug;27(2):55–62. |
| 29 | Vidal K, Sultana S, Patron AP, Salvi I, Shevlyakova M, Foata F, et al. Changing Epidemiology of Acute Respiratory Infections in Under-Two Children in Dhaka, Bangladesh. Front Pediatr. 2022 Jan 10;9:728382. |
| 30 | Juarez SP, Wagner P, Merlo J. Applying measures of discriminatory accuracy to revisit traditional risk factors for being small for gestational age in Sweden: a national cross-sectional study. BMJ Open. 2014;4(7):e005388–e005388. |
| 31 | Kibuchi E, Barua P, Chumo I, Teixeira de Siqueira Filha N, Phillips-Howard P, Mithu MIH, et al. Effects of social determinants on children’s health in informal settlements in Bangladesh and Kenya through an intersectionality lens: a study protocol. BMJ Open. 2022 Jun;12(6):e056494. |
| 32 | Solar O, Irwin A. Commission on Social Determinants of Health : A Conceptual Framework for Action on the Social Determinants of Health [Internet]. 2007 [cited 2021 Jul 18]. Available from: https://www.who.int/social_determinants/resources/csdh_framework_action_05_07.pdf |
| 33 | National Institute of Population Research and Training (NIPORT), MEASURE Evaluation, International Centre for Diarrhoeal Disease Research, Bangladesh (ICDDR,B), Associates for Community and Population Research (ACPR). 2006 Bangladesh Urban Health Survey (UHS). Dhaka, Bangladesh and Chapel Hill, NC, USA: NIPORT, MEASURE Evaluation, ICDDR,B, and ACPR; 2008 p. 1–435. |
| 34 | Filmer D, Pritchett LH. Estimating wealth effects without expenditure data—or tears: An application to educational enrollments in states of India. Demography. 2001;38(1):115–32. |
| 35 | Evans CR, Williams DR, Onnela JP, Subramanian SV. A multilevel approach to modeling health inequalities at the intersection of multiple social identities. Soc Sci Med. 2018;203:64–73. |
| 36 | Evans CR, Leckie G, Merlo J. Multilevel versus single-level regression for the analysis of multilevel information: The case of quantitative intersectional analysis. Social Science & Medicine. 2020;245:112499. |
| 37 | Snijders TAB, Bosker RJ. Multilevel analysis: an introduction to basic and advanced multilevel modeling. 2nd ed. Los Angeles: Sage; 2012. 354 p. |
| 38 | Jaehn P, Mena E, Merz S, Hoffmann R, Gößwald A, Rommel A, et al. Non-response in a national health survey in Germany: An intersectionality-informed multilevel analysis of individual heterogeneity and discriminatory accuracy. PLoS One. 2020;15(8):e0237349. |
| 39 | Holman D, Salway S, Bell A. Mapping intersectional inequalities in biomarkers of healthy ageing and chronic disease in older English adults. Sci Rep. 2020;10(1):13522. |
| 40 | Li Y, Spini D. Intersectional social identities and loneliness: Evidence from a municipality in Switzerland. Journal Community Psychology. 2022 Sep;50(8):3560–73. |
| 41 | Wemrell M, Karlsson N, Perez Vicente R, Merlo J. An intersectional analysis providing more precise information on inequities in self-rated health. International Journal for Equity in Health. 2021 Feb 3;20(1):54. |
| 42 | Goldstein H, Browne W, Rasbash J. Partitioning Variation in Multilevel Models. Understanding Statistics. 2002;1(4):223–31. |
| 43 | Axelsson FS, Mulinari S, Wemrell M, Leckie G, Perez Vicente R, Merlo J. Chronic Obstructive Pulmonary Disease in Sweden: An intersectional multilevel analysis of individual heterogeneity and discriminatory accuracy. SSM Popul Health. 2018;4:334–46. |
| 44 | Wagner P, Merlo J. Discriminatory Accuracy of a Random effect in Multilevel Logistic Regression. International Journal of Epidemiology. 2015;44(suppl_1):i49–50. |
| 45 | Persmark A, Wemrell M, Zettermark S, Leckie G, Subramanian SV, Merlo J. Precision public health: Mapping socioeconomic disparities in opioid dispensations at Swedish pharmacies by Multilevel Analysis of Individual Heterogeneity and Discriminatory Accuracy (MAIHDA). PLoS One. 2019;14(8):1–21. |
| 46 | Zettermark S, Khalaf K, Perez-Vicente R, Leckie G, Mulinari D, Merlo J. Population heterogeneity in associations between hormonal contraception and antidepressant use in Sweden: a prospective cohort study applying intersectional multilevel analysis of individual heterogeneity and discriminatory accuracy (MAIHDA). BMJ Open. 2021 Oct;11(10):e049553. |
| 47 | Merlo J, Chaix B, Ohlsson H, Beckman A, Johnell K, Hjerpe P, et al. A brief conceptual tutorial of multilevel analysis in social epidemiology: using measures of clustering in multilevel logistic regression to investigate contextual phenomena. J Epidemiol Community Health. 2006;60(4):290–7. |
| 48 | Lizotte DJ, Mahendran M, Churchill SM, Bauer GR. Math versus meaning in MAIHDA: A commentary on multilevel statistical models for quantitative intersectionality. Social Science & Medicine. 2020;245:112500. |
| 49 | Carpenter B, Gelman A, Hoffman MD, Lee D, Goodrich B, Betancourt M, et al. *Stan* : A Probabilistic Programming Language. J Stat Soft. 2017;76(1):1–32. |
| 50 | R Core Team T. R: A language and environment for statistical computing. R Foundation for Statistical Computing [Internet]. Vienna, Austria; 2021. Available from: https://www.R-project.org/ |
| 51 | Balloo K, Hosein A, Byrom N, Essau CA. Differences in mental health inequalities based on university attendance: Intersectional multilevel analyses of individual heterogeneity and discriminatory accuracy. SSM - Population Health. 2022 Sep;19:101149. |
| 52 | Taylor ET, Nakai S. Prevalence of Acute Respiratory Infections in Women and Children in Western Sierra Leone due to Smoke from Wood and Charcoal Stoves. IJERPH. 2012 Jun 19;9(6):2252–65. |
| 53 | Adams AM, Islam R, Ahmed T. Who serves the urban poor? A geospatial and descriptive analysis of health services in slum settlements in Dhaka, Bangladesh. Health Policy and Planning. 2015 Mar 1;30(suppl 1):i32–45. |
| 54 | Gupta RPS, de Wit ML, McKeown D. The impact of poverty on the current and future health status of children. Paediatrics & Child Health. 2007 Oct;12(8):667–72. |
| 55 | Kamal MM, Hasan MM, Davey R. Determinants of childhood morbidity in Bangladesh: evidence from the Demographic and Health Survey 2011. BMJ Open. 2015 Oct;5(10):e007538. |
| 56 | Mulatya DM, Mutuku FW. Assessing Comorbidity of Diarrhea and Acute Respiratory Infections in Children Under 5 Years: Evidence From Kenya’s Demographic Health Survey 2014. J Prim Care Community Health. 2020 Jan;11:215013272092519. |
| 57 | Siziya S, Muula A, Rudatsikira E. Correlates of diarrhoea among children below the age of 5 years in Sudan. Afr H Sci. 2013 Sep 5;13(2):376–83. |
| 58 | Alam MA, Hakim M, Rouf M, Haque MO, Ali M, Zaidul I. Nutritional status of urban slum children below five years: Assessment by anthropometric measurements with special reference to socioeconomic status. J Food Agr Environ. 2011;9(2):85–90. |
| 59 | Rahman A, Chowdhury S, Hossain D. Acute Malnutrition in Bangladeshi Children: Levels and Determinants. Asia Pac J Public Health. 2009;21(3):294–302. |
| 60 | Khan J, Awan N. A comprehensive analysis on child mortality and its determinants in Bangladesh using frailty models. Archives of Public Health. 2017;75. |
| 61 | Gutberlet J, Uddin SMN. Household waste and health risks affecting waste pickers and the environment in low- and middle-income countries. International Journal of Occupational and Environmental Health. 2017 Oct 2;23(4):299–310. |
| 62 | Zethelius B, Berglund L, Sundström J, Ingelsson E, Basu S, Larsson A, et al. Use of Multiple Biomarkers to Improve the Prediction of Death from Cardiovascular Causes. N Engl J Med. 2008 May 15;358(20):2107–16. |
| 63 | Cooney MT, Dudina AL, Graham IM. Value and Limitations of Existing Scores for the Assessment of Cardiovascular Risk. Journal of the American College of Cardiology. 2009 Sep;54(14):1209–27. |
| 64 | Melander O. Novel and Conventional Biomarkers for Prediction of Incident Cardiovascular Events in the Community. JAMA. 2009 Jul 1;302(1):49. |
| 65 | Vlahov D. Urban Health in Bangladesh. In: Hossain MM, Majumder MAH, Chin B, Islam MZ, Adams AM, editors. Slum health in Bangladesh: insights for health and demographic surveillance. Dhaka: Health Systems and Population Studies Division, icddr,b; 2019. (Special publication / International Centre for Diarrhoeal Disease Research, Bangladesh). |
| 66 | Kang H. The prevention and handling of the missing data. Korean J Anesthesiol. 2013;64(5):402. |

***Before revision***

| **Ref #** | **Reference style: Vancouver** |
| --- | --- |
| 1 | Hossain B. Slum of Bangladesh: An Overview [Internet]. 2020 [cited 2021 Dec 6]. Available from: https://www.researchgate.net/publication/339569720_Slum_of_Bangladesh_An_Overview |
| 2 | Islam N, Mahbub A, Islam N. Urban slums of Bangladesh [Internet]. The Daily Star. 2009 [cited 2021 Dec 4]. Available from: https://www.thedailystar.net/news-detail-93293 |
| 3 | BBS. Preliminary Report on the Census of Slum Areas and Floating Population 2014 [Internet]. Bangladesh Bureau of Statistics (BBS), Statistics and Informatics Division (SID), Ministry of Planning Government of the People’s Republic of Bangladesh; 2015 [cited 2021 Dec 17]. Available from: http://203.112.218.65:8008/WebTestApplication/userfiles/Image/Slum/Preli_Slum_Census.pdf |
| 4 | NIPORT, icddr,b, MEASURE Evaluation. Bangladesh Urban Health Survey 2013 Final Report — MEASURE Evaluation [Internet]. 2015 [cited 2021 Jul 9]. Available from: https://www.measureevaluation.org/resources/publications/tr-15-117.html |
| 5 | Latif MB, Irin A, Ferdaus J. Socio-economic and health status of slum dwellers of the Kalyanpur slum in Dhaka city. Bangladesh J Sci Res [Internet]. 2016 [cited 2021 Dec 22];29(1):73–83. Available from: https://www.banglajol.info/index.php/BJSR/article/view/29760 |
| 6 | Bangladesh Bureau of Statistics, UNICEF Bangladesh, editors. Child well-being survey in urban areas of Bangladesh 2016: final report. Dhaka, Bangladesh: Bangladesh Bureau of Statistics and UNICEF Bangladesh; 2016. 152 p. |
| 7 | Islam MM, Debnath J, Rakhi NN, Al-Jubayer A. Epidemiologic pattern of respiratory diseases among hospitalized pediatric patients in Khulna, Bangladesh. Int J Sci Rep [Internet]. 2021 [cited 2022 Jun 13];7(6):306. Available from: https://www.sci-rep.com/index.php/scirep/article/view/929 |
| 8 | Rahman A, Hossain MdM. Prevalence and determinants of fever, ARI and diarrhea among children aged 6–59 months in Bangladesh. BMC Pediatr [Internet]. 2022 [cited 2022 Jun 6];22(1):117. Available from: https://bmcpediatr.biomedcentral.com/articles/10.1186/s12887-022-03166-9 |
| 9 | Mberu BU, Haregu TN, Kyobutungi C, Ezeh AC. Health and health-related indicators in slum, rural, and urban communities: a comparative analysis. Global Health Action [Internet]. 2016 [cited 2022 Jan 2];9(1):33163. Available from: https://www.tandfonline.com/doi/full/10.3402/gha.v9.33163 |
| 10 | Zanuzdana A, Khan MMH, Brinkel JK, Kraemer A. Factors associated with high prevalence of coughs, colds and fever among urban slum dwellers in Dhaka, Bangladesh. SE Asia J Pub Health [Internet]. 2014 [cited 2022 Apr 17];3(2):58–65. Available from: https://www.banglajol.info/index.php/SEAJPH/article/view/20042 |
| 11 | Sayem AM, Nury ATMdS, Hossain MdD. Achieving the Millennium Development Goal for Under-five Mortality in Bangladesh: Current Status and Lessons for Issues and Challenges for Further Improvements. J Health Popul Nutr [Internet]. 2011 Apr [cited 2022 Oct 18];29(2):92–102. Available from: https://www.ncbi.nlm.nih.gov/pmc/articles/PMC3126981/ |
| 12 | Dewey KG, Mayers DR. Early child growth: how do nutrition and infection interact?: How do nutrition and infection interact? Maternal & Child Nutrition [Internet]. 2011 Oct [cited 2022 Jul 25];7:129–42. Available from: https://onlinelibrary.wiley.com/doi/10.1111/j.1740-8709.2011.00357.x |
| 13 | Biermann O, Mwoka M, Ettman CK, Abdalla SM, Shawky S, Ambuko J, et al. Data, Social Determinants, and Better Decision-making for Health: the 3-D Commission. J Urban Health [Internet]. 2021 [cited 2022 Jun 12];98(S1):4–14. Available from: https://link.springer.com/10.1007/s11524-021-00556-9 |
| 14 | Kundu S, Kundu S, Banna MdHA, Ahinkorah BO, Seidu AA, Okyere J. Prevalence and factors associated with childhood diarrheal disease and acute respiratory infection in Bangladesh: An analysis of a nationwide cross-sectional survey [Internet]. In Review; 2021 [cited 2022 Jun 14]. Available from: https://www.researchsquare.com/article/rs-717076/v1 |
| 15 | Ullah MB, Mridha MK, Arnold CD, Matias SL, Khan MSA, Siddiqui Z, et al. Factors associated with diarrhea and acute respiratory infection in children under two years of age in rural Bangladesh. BMC Pediatr [Internet]. 2019 [cited 2022 Jun 28];19(1):386. Available from: https://bmcpediatr.biomedcentral.com/articles/10.1186/s12887-019-1738-6 |
| 16 | World Health Organization. A conceptual framework for action on the social determinants of health. Social Determinants of Health Discussion Paper 2 (Policy and Practice) [Internet]. 2010 [cited 2021 Jul 18]. Available from: http://apps.who.int/iris/bitstream/10665/44489/1/9789241500852_eng.pdf |
| 17 | Ahmed I. Factors in building resilience in urban slums of Dhaka, Bangladesh. Procedia Economics and Finance [Internet]. 2014 [cited 2022 Jan 4];18:745–53. Available from: https://www.sciencedirect.com/science/article/pii/S2212567114009988 |
| 18 | Perry H, Weierbach R, Hossain I, Islam R. Childhood immunization coverage in zone 3 of Dhaka City: the challenge of reaching impoverished households in urban Bangladesh. Bull World Health Organ. 1998;76(6):565–73. |
| 19 | Tarlov AR. Social determinants of health: The sociobiological translation. In: Health and Social Organization [Internet]. 0 ed. Routledge; 2002 [cited 2021 Jul 18]. p. 87–109. Available from: https://www.taylorfrancis.com/books/9781134791118/chapters/10.4324/9780203434932-11 |
| 20 | Dovey K, King R. Forms of Informality: Morphology and Visibility of Informal Settlements. Built Environment (1978-) [Internet]. 2011 [cited 2021 Jul 18];37(1):11–29. Available from: http://www.jstor.org/stable/23289768 |
| 21 | World Health Organization. A billion voices: listening and responding to the health needs of slum dwellers and informal settlers in new urban settings [Internet]. WHO Kobe: Japan; 2005. Available from: https://www.who.int/social_determinants/resources/urban_settings.pdf |
| 22 | Heard E, Fitzgerald L, Wigginton B, Mutch A. Applying intersectionality theory in health promotion research and practice. Health Promotion International [Internet]. 2020 Aug 1 [cited 2022 Nov 25];35(4):866–76. Available from: https://academic.oup.com/heapro/article/35/4/866/5544760 |
| 23 | Merlo J. Multilevel analysis of individual heterogeneity and discriminatory accuracy (MAIHDA) within an intersectional framework. Soc Sci Med. 2018;203:74–80. |
| 24 | Zahan N, Sayed A, Mamun M, Sabiruzzaman N, Islam, Hossain A, et al. Factors Associated with Acute Respiratory Infections among Under-Five Children in Bangladesh. International Journal of Statistical Sciences. 2016 Jan 1;15(Special Issue):85–100. |
| 25 | Sultana M, Sarker AR, Sheikh N, Akram R, Ali N, Mahumud RA, et al. Prevalence, determinants and health care-seeking behavior of childhood acute respiratory tract infections in Bangladesh. PLoS ONE [Internet]. 2019 [cited 2022 Jun 14];14(1):1–18. Available from: https://dx.plos.org/10.1371/journal.pone.0210433 |
| 26 | Mostafa I, Naila NN, Mahfuz M, Roy M, Faruque ASG, Ahmed T. Children living in the slums of Bangladesh face risks from unsafe food and water and stunted growth is common. Acta Paediatr [Internet]. 2018 Jul [cited 2022 Jul 20];107(7):1230–9. Available from: https://onlinelibrary.wiley.com/doi/10.1111/apa.14281 |
| 27 | Yaya S, Bishwajit G. Burden of Acute Respiratory Infections Among Under-Five Children in Relation to Household Wealth and Socioeconomic Status in Bangladesh. TropicalMed [Internet]. 2019 Feb 12 [cited 2022 Jun 28];4(1):36. Available from: http://www.mdpi.com/2414-6366/4/1/36 |
| 28 | Rahman MM, Shahidullah M. Risk factors for acute respiratory infections among the slum infants of Dhaka city. Bangladesh Med Res Counc Bull [Internet]. 2001 Aug;27(2):55–62. Available from: https://bmrcbulletin.org.bd/wp-content/uploads/2021/09/MM-Rahman-Page-55-62.pdf |
| 29 | Vidal K, Sultana S, Patron AP, Salvi I, Shevlyakova M, Foata F, et al. Changing Epidemiology of Acute Respiratory Infections in Under-Two Children in Dhaka, Bangladesh. Front Pediatr [Internet]. 2022 Jan 10 [cited 2022 Jul 21];9:728382. Available from: https://www.frontiersin.org/articles/10.3389/fped.2021.728382/full |
| 30 | Juarez SP, Wagner P, Merlo J. Applying measures of discriminatory accuracy to revisit traditional risk factors for being small for gestational age in Sweden: a national cross-sectional study. BMJ Open [Internet]. 2014 [cited 2022 Jun 14];4(7):e005388–e005388. Available from: https://bmjopen.bmj.com/lookup/doi/10.1136/bmjopen-2014-005388 |
| 31 | Kibuchi E, Barua P, Chumo I, Teixeira de Siqueira Filha N, Phillips-Howard P, Mithu MIH, et al. Effects of social determinants on children’s health in informal settlements in Bangladesh and Kenya through an intersectionality lens: a study protocol. BMJ Open [Internet]. 2022 Jun [cited 2022 Jun 12];12(6):e056494. Available from: https://bmjopen.bmj.com/lookup/doi/10.1136/bmjopen-2021-056494 |
| 32 | Solar O, Irwin A. Commission on Social Determinants of Health : A Conceptual Framework for Action on the Social Determinants of Health [Internet]. 2007 [cited 2021 Jul 18]. Available from: https://www.who.int/social_determinants/resources/csdh_framework_action_05_07.pdf |
| 33 | Angeles G, Al-Sabir A, Lance P, Buckner B, Streatfield PK, Karar ZA, et al. 2006 Bangladesh Urban Health Survey (UHS) — MEASURE Evaluation [Internet]. 2008 [cited 2021 Dec 2]. Available from: https://www.measureevaluation.org/resources/publications/tr-08-68.html |
| 34 | Filmer D, Pritchett LH. Estimating wealth effects without expenditure data—or tears: An application to educational enrollments in states of India. Demography [Internet]. 2001 [cited 2022 Jun 6];38(1):115–32. Available from: https://read.dukeupress.edu/demography/article/38/1/115/170414/Estimating-wealth-effects-without-expenditure-data |
| 35 | Evans CR, Williams DR, Onnela JP, Subramanian SV. A multilevel approach to modeling health inequalities at the intersection of multiple social identities. Soc Sci Med. 2018;203:64–73. |
| 36 | Evans CR, Leckie G, Merlo J. Multilevel versus single-level regression for the analysis of multilevel information: The case of quantitative intersectional analysis. Social Science & Medicine [Internet]. 2020 [cited 2021 Dec 3];245:112499. Available from: https://linkinghub.elsevier.com/retrieve/pii/S0277953619304927 |
| 37 | Jaehn P, Mena E, Merz S, Hoffmann R, Gößwald A, Rommel A, et al. Non-response in a national health survey in Germany: An intersectionality-informed multilevel analysis of individual heterogeneity and discriminatory accuracy. PLoS One. 2020;15(8):e0237349. |
| 38 | Snijders TAB, Bosker RJ. Multilevel analysis: an introduction to basic and advanced multilevel modeling. 2nd ed. Los Angeles: Sage; 2012. 354 p. |
| 39 | Holman D, Salway S, Bell A. Mapping intersectional inequalities in biomarkers of healthy ageing and chronic disease in older English adults. Sci Rep [Internet]. 2020 [cited 2022 Jan 20];10(1):13522. Available from: https://www.nature.com/articles/s41598-020-69934-8 |
| 40 | Li Y, Spini D. Intersectional social identities and loneliness: Evidence from a municipality in Switzerland. Journal Community Psychology [Internet]. 2022 Sep [cited 2022 Nov 26];50(8):3560–73. Available from: https://onlinelibrary.wiley.com/doi/10.1002/jcop.22855 |
| 41 | Wemrell M, Karlsson N, Perez Vicente R, Merlo J. An intersectional analysis providing more precise information on inequities in self-rated health. International Journal for Equity in Health [Internet]. 2021 Feb 3 [cited 2022 Nov 26];20(1):54. Available from: https://doi.org/10.1186/s12939-020-01368-0 |
| 42 | Jaehn P, Mena E, Merz S, Hoffmann R, Gößwald A, Rommel A, et al. Non-response in a national health survey in Germany: An intersectionality-informed multilevel analysis of individual heterogeneity and discriminatory accuracy. PLoS One. 2020;15(8):e0237349. |
| 43 | Goldstein H, Browne W, Rasbash J. Partitioning Variation in Multilevel Models. Understanding Statistics [Internet]. 2002 [cited 2021 Dec 3];1(4):223–31. Available from: http://www.tandfonline.com/doi/abs/10.1207/S15328031US0104_02 |
| 44 | Fisk SA, Mulinari S, Wemrell M, Leckie G, Vicente RP, Merlo J. Chronic Obstructive Pulmonary Disease in Sweden: An intersectional multilevel analysis of individual heterogeneity and discriminatory accuracy. SSM - population health. 2018; |
| 45 | Persmark A, Wemrell M, Zettermark S, Leckie G, Subramanian SV, Merlo J. Precision public health: Mapping socioeconomic disparities in opioid dispensations at Swedish pharmacies by Multilevel Analysis of Individual Heterogeneity and Discriminatory Accuracy (MAIHDA). PLoS One [Internet]. 2019;14(10):e0224008. Available from: https://journals.plos.org/plosone/article?id=10.1371/journal.pone.0220322 |
| 46 | Wagner P, Merlo J. Discriminatory Accuracy of a Random effect in Multilevel Logistic Regression. International Journal of Epidemiology [Internet]. 2015 [cited 2021 Dec 30];44(suppl_1):i49–50. Available from: https://academic.oup.com/ije/article/2573789/Discriminatory |
| 47 | Zettermark S, Khalaf K, Perez-Vicente R, Leckie G, Mulinari D, Merlo J. Population heterogeneity in associations between hormonal contraception and antidepressant use in Sweden: a prospective cohort study applying intersectional multilevel analysis of individual heterogeneity and discriminatory accuracy (MAIHDA). BMJ Open [Internet]. 2021 Oct [cited 2022 Jul 18];11(10):e049553. Available from: https://bmjopen.bmj.com/lookup/doi/10.1136/bmjopen-2021-049553 |
| 48 | Merlo J, Chaix B, Ohlsson H, Beckman A, Johnell K, Hjerpe P, et al. A brief conceptual tutorial of multilevel analysis in social epidemiology: using measures of clustering in multilevel logistic regression to investigate contextual phenomena. J Epidemiol Community Health. 2006;60(4):290–7. |
| 49 | Lizotte DJ, Mahendran M, Churchill SM, Bauer GR. Math versus meaning in MAIHDA: A commentary on multilevel statistical models for quantitative intersectionality. Social Science & Medicine [Internet]. 2020 [cited 2022 Jan 8];245:112500. Available from: https://doi.org/10.1016/j.socscimed.2019.112500 |
| 50 | Carpenter B, Gelman A, Hoffman MD, Lee D, Goodrich B, Betancourt M, et al. *Stan* : A Probabilistic Programming Language. J Stat Soft [Internet]. 2017 [cited 2021 Dec 3];76(1). Available from: http://www.jstatsoft.org/v76/i01/ |
| 51 | Axelsson FS, Mulinari S, Wemrell M, Leckie G, Perez Vicente R, Merlo J. Chronic Obstructive Pulmonary Disease in Sweden: An intersectional multilevel analysis of individual heterogeneity and discriminatory accuracy. SSM Popul Health. 2018;4:334–46. |
| 52 | R Core Team T. R: A language and environment for statistical computing. R Foundation for Statistical Computing [Internet]. Vienna, Austria; 2021. Available from: https://www.R-project.org/ |
| 53 | Balloo K, Hosein A, Byrom N, Essau CA. Differences in mental health inequalities based on university attendance: Intersectional multilevel analyses of individual heterogeneity and discriminatory accuracy. SSM - Population Health [Internet]. 2022 Sep [cited 2022 Jul 20];19:101149. Available from: https://doi.org/10.1016/j.ssmph.2022.101149 |
| 54 | Taylor ET, Nakai S. Prevalence of Acute Respiratory Infections in Women and Children in Western Sierra Leone due to Smoke from Wood and Charcoal Stoves. IJERPH [Internet]. 2012 Jun 19 [cited 2022 Oct 8];9(6):2252–65. Available from: http://www.mdpi.com/1660-4601/9/6/2252 |
| 55 | Adams AM, Islam R, Ahmed T. Who serves the urban poor? A geospatial and descriptive analysis of health services in slum settlements in Dhaka, Bangladesh. Health Policy and Planning [Internet]. 2015 Mar 1 [cited 2022 Jul 25];30(suppl 1):i32–45. Available from: https://academic.oup.com/heapol/article-lookup/doi/10.1093/heapol/czu094 |
| 56 | Gupta RPS, de Wit ML, McKeown D. The impact of poverty on the current and future health status of children. Paediatrics & Child Health [Internet]. 2007 Oct [cited 2022 Oct 9];12(8):667–72. Available from: https://academic.oup.com/pch/article-lookup/doi/10.1093/pch/12.8.667 |
| 57 | Kamal MM, Hasan MM, Davey R. Determinants of childhood morbidity in Bangladesh: evidence from the Demographic and Health Survey 2011. BMJ Open [Internet]. 2015 Oct [cited 2022 Oct 9];5(10):e007538. Available from: https://bmjopen.bmj.com/lookup/doi/10.1136/bmjopen-2014-007538 |
| 58 | Mulatya DM, Mutuku FW. Assessing Comorbidity of Diarrhea and Acute Respiratory Infections in Children Under 5 Years: Evidence From Kenya’s Demographic Health Survey 2014. J Prim Care Community Health [Internet]. 2020 Jan [cited 2022 Oct 9];11:215013272092519. Available from: http://journals.sagepub.com/doi/10.1177/2150132720925190 |
| 59 | Siziya S, Muula A, Rudatsikira E. Correlates of diarrhoea among children below the age of 5 years in Sudan. Afr H Sci [Internet]. 2013 Sep 5 [cited 2022 Oct 9];13(2):376–83. Available from: http://www.ajol.info/index.php/ahs/article/view/93525 |
| 60 | Alam MA, Hakim M, Rouf M, Haque MO, Ali M, Zaidul I. Nutritional status of urban slum children below five years: Assessment by anthropometric measurements with special reference to socioeconomic status. J Food Agr Environ. 2011;9(2):85–90. |
| 61 | Rahman A, Chowdhury S, Hossain D. Acute Malnutrition in Bangladeshi Children: Levels and Determinants. Asia Pac J Public Health [Internet]. 2009 [cited 2022 Jan 8];21(3):294–302. Available from: http://journals.sagepub.com/doi/10.1177/1010539509335399 |
| 62 | Khan J, Awan N. A comprehensive analysis on child mortality and its determinants in Bangladesh using frailty models. Archives of Public Health. 2017;75. |
| 63 | Gutberlet J, Uddin SMN. Household waste and health risks affecting waste pickers and the environment in low- and middle-income countries. International Journal of Occupational and Environmental Health [Internet]. 2017 Oct 2 [cited 2022 Oct 5];23(4):299–310. Available from: https://www.tandfonline.com/doi/full/10.1080/10773525.2018.1484996 |
| 64 | Zethelius B, Berglund L, Sundström J, Ingelsson E, Basu S, Larsson A, et al. Use of Multiple Biomarkers to Improve the Prediction of Death from Cardiovascular Causes. N Engl J Med [Internet]. 2008 May 15 [cited 2022 Jul 21];358(20):2107–16. Available from: http://www.nejm.org/doi/abs/10.1056/NEJMoa0707064 |
| 65 | Cooney MT, Dudina AL, Graham IM. Value and Limitations of Existing Scores for the Assessment of Cardiovascular Risk. Journal of the American College of Cardiology [Internet]. 2009 Sep [cited 2022 Jul 21];54(14):1209–27. Available from: https://linkinghub.elsevier.com/retrieve/pii/S0735109709025029 |
| 66 | Melander O. Novel and Conventional Biomarkers for Prediction of Incident Cardiovascular Events in the Community. JAMA [Internet]. 2009 Jul 1 [cited 2022 Jul 21];302(1):49. Available from: http://jama.jamanetwork.com/article.aspx?doi=10.1001/jama.2009.943 |
| 67 | Vlahov D. Urban Health in Bangladesh. In: Hossain MM, Majumder MAH, Chin B, Islam MZ, Adams AM, editors. Slum health in Bangladesh: insights for health and demographic surveillance. Dhaka: Health Systems and Population Studies Division, icddr,b; 2019. (Special publication / International Centre for Diarrhoeal Disease Research, Bangladesh). |
| 68 | Kang H. The prevention and handling of the missing data. Korean J Anesthesiol [Internet]. 2013 [cited 2022 Jul 24];64(5):402. Available from: http://ekja.org/journal/view.php?doi=10.4097/kjae.2013.64.5.402 |

2. Please insert an Ethics Statement at the beginning of your Methods section, under a subheading 'Ethics Statement'. It must include:

1) The name(s) of the Institutional Review Board(s) or Ethics Committee(s)

2) The approval number(s), or a statement that approval was granted by the named board(s)

3) (for human participants/donors) - A statement that formal consent was obtained (must state whether verbal/written) OR the reason consent was not obtained (e.g. anonymity). NOTE: If child participants, the statement must declare that formal consent was obtained from the parent/guardian.

- **Thank you for your suggestion. We have inserted Ethics Statement at the beginning of the Methods section, under a subheading ‘Ethics Statement’ as follows.**
- ***“Ethics Statement***

*We used secondary deidentified data set. Ethical approval was not required for the study. The dataset named “Bangladesh Urban Health Survey (UHS) 2013” which was used in this study is publicly available at the Website (*[***https://dataverse.unc.edu/dataset.xhtml?persistentId=doi:10.15139/S3/12274***](https://dataverse.unc.edu/dataset.xhtml?persistentId=doi:10.15139/S3/12274)*) upon responsible request.* For UHS 2013, ethical approval was obtained from the Bangladesh Medical Research Council and the Institutional Review Board at the School of Public Health, University of North Carolina at Chapel Hill (NIPORT et al., 2015).”

**Reference:**

NIPORT, icddr,b, MEASURE Evaluation. Bangladesh Urban Health Survey 2013 Final Report. Chapel Hill, USA: NIPORT, ICDDR,B and MEASURE Evaluation; 2015 p. 1–219.

3. Fig 4: please (a) provide a direct link to the base layer of the map (i.e., the country or region border shape) and ensure this is also included in the figure legend; and (b) provide a link to the terms of use / license information for the base layer image or shapefile. We cannot publish proprietary or copyrighted maps (e.g. Google Maps, Mapquest) and the terms of use for your map base layer must be compatible with our CC-BY 4.0 license.

Note: if you created the map in a software program like R or ArcGIS, please locate and indicate the source of the basemap shapefile onto which data has been plotted.

If your map was obtained from a copyrighted source please amend the figure so that the base map used is from an openly available source. Alternatively, please provide explicit written permission from the copyright holder granting you the right to publish the material under our CC-BY 4.0 license.

Please note that the following CC BY licenses are compatible with PLOS license: CC BY 4.0, CC BY 2.0 and CC BY 3.0, meanwhile such licenses as CC BY-ND 3.0 and others are not compatible due to additional restrictions.

If you are unsure whether you can use a map or not, please do reach out and we will be able to help you. The following websites are good examples of where you can source open access or public domain maps: 
* U.S. Geological Survey (USGS) - All maps are in the public domain. (http://www.usgs.gov) 
* PlaniGlobe - All maps are published under a Creative Commons license so please cite “PlaniGlobe, http://www.planiglobe.com, CC BY 2.0” in the image credit after the caption. (http://www.planiglobe.com/?lang=enl) 
* Natural Earth - All maps are public domain. (<http://www.naturalearthdata.com/about/terms-of-use/>)

- ***Thank you We have provided additional information in the revised manuscript as requested.***
- ***(a) R software was used to create the map. the source of the basemap shapefile is available:*** [***https://geodata.ucdavis.edu/gadm/gadm4.1/shp/gadm41_BGD_shp.zip***](https://geodata.ucdavis.edu/gadm/gadm4.1/shp/gadm41_BGD_shp.zip)

***(b) The data are freely available for academic use and other non-commercial use (CC-BY 2.0).*** [***https://gadm.org/license.html***](https://gadm.org/license.html)

- ***We have added one more map (Fig 1) and rearranged figures in the revised manuscript. Fig 4 is now Fig 5 in the revised manuscript.***

**Fig 1. Study area of Bangladesh Urban Health Survey 2013 (UHS 2013)**

**This is the Fig 1 legend.**

**UHS 2013 has been conducted in seven divisions of Bangladesh:** Barisal, Chittagong, Dhaka, Khulna, Rajshahi, Rangpur and Sylhet.

R software was used to create the map. The source of the basemap shapefile is available on GADM website at: <https://geodata.ucdavis.edu/gadm/gadm4.1/shp/gadm41_BGD_shp.zip>. The data are freely available for academic use and other non-commercial use (CC-BY 2.0). The GADM license is available at <https://gadm.org/license.html>

Reviewers' comments:

Reviewer's Responses to Questions

**Comments to the Author**

1. If the authors have adequately addressed your comments raised in a previous round of review and you feel that this manuscript is now acceptable for publication, you may indicate that here to bypass the “Comments to the Author” section, enter your conflict of interest statement in the “Confidential to Editor” section, and submit your "Accept" recommendation.

Reviewer #1: (No Response)

Reviewer #2: All comments have been addressed

2. Does this manuscript meet PLOS Global Public Health’s [publication criteria](https://journals.plos.org/globalpublichealth/s/criteria-for-publication)? Is the manuscript technically sound, and do the data support the conclusions? The manuscript must describe methodologically and ethically rigorous research with conclusions that are appropriately drawn based on the data presented.

Reviewer #1: Yes

Reviewer #2: Yes

3. Has the statistical analysis been performed appropriately and rigorously?

Reviewer #1: Yes

Reviewer #2: Yes

4. Have the authors made all data underlying the findings in their manuscript fully available (please refer to the Data Availability Statement at the start of the manuscript PDF file)?

The [PLOS Data policy](https://journals.plos.org/globalpublichealth/s/data-availability) requires authors to make all data underlying the findings described in their manuscript fully available without restriction, with rare exception. The data should be provided as part of the manuscript or its supporting information, or deposited to a public repository. For example, in addition to summary statistics, the data points behind means, medians and variance measures should be available. If there are restrictions on publicly sharing data—e.g. participant privacy or use of data from a third party—those must be specified.

Reviewer #1: Yes

Reviewer #2: Yes

5. Is the manuscript presented in an intelligible fashion and written in standard English?

PLOS Global Public Health does not copyedit accepted manuscripts, so the language in submitted articles must be clear, correct, and unambiguous. Any typographical or grammatical errors should be corrected at revision, so please note any specific errors here.

Reviewer #1: No

Reviewer #2: Yes

**6. Review Comments to the Author**

Please use the space provided to explain your answers to the questions above. You may also include additional comments for the author, including concerns about dual publication, research ethics, or publication ethics. (Please upload your review as an attachment if it exceeds 20,000 characters)

**Reviewer #1:**

1. Thank you for the edits and thoughtful responses to my initial comments. I believe you have addressed the majority of my questions; however, the text still requires copyediting for grammar. A few instances from the introduction are listed below, though this is not comprehensive as copyediting is not the specific role of a reviewer. Some common errors noted are missing articles (for example, "the"), plural vs singular conjugation, and verb-tense agreement. Would recommend a *detailed* review specifically for grammar.

- ***Thank you for reviewing the revised manuscript and we greatly appreciate your valuable time and effort. We have addressed all comments and suggestions in the revised manuscript. We have thoroughly reviewed and made necessary corrections in the revised manuscript.***

Line 59: "In Bangladesh context, most widely accepted definition..." should read "In the Bangladeshi context, the most widely accepted definition..."

- ***Done***

Line 63: should read, "according to the Census of..."

- ***Done***

Line 69: should read, "there is also regional variation in [prevalence? incidence?] of childhood illness..."

- ***Done***

  Line 74: should read, "among males compared to females"
- ***Done***

Line 81: "in future" should be "in the future"

- ***Done***

Line 96: should read "they lead to aggravated social inequities"

- ***Done***

2. I appreciate the addition of the map. There is a typo in the "reference division" label that should be corrected.

- ***Thank you for correcting the critical mistakes. We have revised the map in the revised manuscript (Fig 5). We have added these texts in the revised manuscript “The combined divisions of Barisal, Chittagong, Rangpur and Sylhet had higher risk of cough, fever and ARI than those living in Dhaka Division. Similarly, U5y living in Rajshahi and Khulna divisions were more likely to have cough and ARI, respectively, compared to those living in slums in in Dhaka Division”***

**Fig 5. Health outcomes of children under five years old (U5y) living in slums in different divisions of Bangladesh**

**This is the Fig 5 legend.**

R software was used to create the map. The source of the basemap shapefile is available on GADM website at: <https://geodata.ucdavis.edu/gadm/gadm4.1/shp/gadm41_BGD_shp.zip>. The data are freely available for academic use and other non-commercial use (CC-BY 2.0). The GADM license is available at <https://gadm.org/license.html>

**Reviewer #2:**

Thank you for addressing the substantive comments. I do have reservations about picking variables based on statistical significance but I understand if that is consistent with practice in this line of the literature. "It was not possible to include all variables because our analyses included main effects that only showed statistical significance (a p-value less than 0.05) on univariate analysis" (page 9 of the manuscript) is circular because that was a decision you made in the first place. Perhaps a better justification would be that you wanted to keep the analysis tractable.

- ***Thank you for reviewing the revised manuscript and we greatly appreciate your valuable time and effort. We have addressed your comments and suggestions in the revised manuscript. We have added these texts in the revised manuscript “However, we have selected variables based on statistical significance to keep the analysis tractable ”***

7. PLOS authors have the option to publish the peer review history of their article ([what does this mean?](https://journals.plos.org/globalpublichealth/s/editorial-and-peer-review-process#loc-peer-review-history)). If published, this will include your full peer review and any attached files.


**Do you want your identity to be public for this peer review?** If you choose “no”, your identity will remain anonymous but your review may still be made public.


For information about this choice, including consent withdrawal, please see our [Privacy Policy](https://www.plos.org/privacy-policy).

Reviewer #1: No

Reviewer #2: No
